# Single-cell RNA-Seq analysis reveals cell subsets and gene signatures associated with rheumatoid arthritis disease activity

**DOI:** 10.1172/jci.insight.178499

**Published:** 2024-07-02

**Authors:** Marie Binvignat, Brenda Y. Miao, Camilla Wibrand, Monica M. Yang, Dmitry Rychkov, Emily Flynn, Joanne Nititham, Whitney Tamaki, Umair Khan, Alexander Carvidi, Melissa Krueger, Erene Niemi, Yang Sun, Gabriela K. Fragiadakis, Jérémie Sellam, Encarnita Mariotti-Ferrandiz, David Klatzmann, Andrew J. Gross, Chun Jimmie Ye, Atul J. Butte, Lindsey A. Criswell, Mary C. Nakamura, Marina Sirota

**Affiliations:** 1 Bakar Computational Health Sciences Institute, UCSF, San Francisco, California, USA.; 2 Immunology Immunopathology Immunotherapy, Pitie Salpetriere Hospital UMRS 959, Sorbonne University, Paris, France.; 3 Department of Rheumatology, Research Center Saint Antoine, UMRS 938, Sorbonne University, AP-HP, Saint-Antoine Hospital, Inserm UMRS 938, Paris, France.; 4 Aarhus University, Aarhus, Denmark.; 5 Rosalind Russell/Ephraim P. Engleman Rheumatology Research Center, Division of Rheumatology, Department of Medicine, and; 6 CoLabs, UCSF, San Francisco, California, USA.; 7 Department of Medicine, Oregon Health & Science University, Portland, Oregon, USA.; 8 Department of Human Genetics and; 9 Department of Pediatrics, UCSF, San Francisco, California, USA.; 10 National Human Genome Research Institute (NHGRI), NIH, Bethesda, Maryland, USA.; 11 San Francisco VA Health Care System, San Francisco, California, USA.

**Keywords:** Autoimmunity, Immunology, Rheumatology

## Abstract

Rheumatoid arthritis (RA) management leans toward achieving remission or low disease activity. In this study, we conducted single-cell RNA sequencing (scRNA-Seq) of peripheral blood mononuclear cells (PBMCs) from 36 individuals (18 patients with RA and 18 matched controls, accounting for age, sex, race, and ethnicity), to identify disease-relevant cell subsets and cell type–specific signatures associated with disease activity. Our analysis revealed 18 distinct PBMC subsets, including an IFN-induced transmembrane 3–overexpressing (IFITM3-overexpressing) IFN-activated monocyte subset. We observed an increase in CD4^+^ T effector memory cells in patients with moderate-high disease activity (DAS28-CRP ≥ 3.2) and a decrease in nonclassical monocytes in patients with low disease activity or remission (DAS28-CRP < 3.2). Pseudobulk analysis by cell type identified 168 differentially expressed genes between RA and matched controls, with a downregulation of proinflammatory genes in the γδ T cell subset, alteration of genes associated with RA predisposition in the IFN-activated subset, and nonclassical monocytes. Additionally, we identified a gene signature associated with moderate-high disease activity, characterized by upregulation of proinflammatory genes such as *TNF*, *JUN*, *EGR1*, *IFIT2*, *MAFB*, and *G0S2* and downregulation of genes including *HLA-DQB1*, *HLA-DRB5*, and *TNFSF13B*. Notably, cell-cell communication analysis revealed an upregulation of signaling pathways, including VISTA, in both moderate-high and remission-low disease activity contexts. Our findings provide valuable insights into the systemic cellular and molecular mechanisms underlying RA disease activity.

## Abstract 2

Identifying new cell subsets, genes and cell-cell communications associated with disease activity in Rheumatoid Athritis using singel cell RNA sequencing

## Graphical abstract

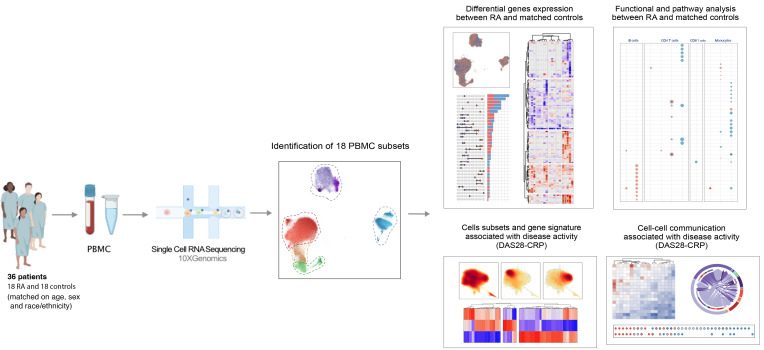

## Introduction

Rheumatoid arthritis (RA) is a systemic autoimmune disease characterized by chronic inflammation and joint destruction (1). While the prevalence and disease burden vary considerably between geographic regions and populations (2), RA affects approximately 1.3 million adults in the United States, representing 0.6%–1% of the country’s population (3, 4). RA is a debilitating condition and a major socioeconomic burden, with a prevalence of work disability around 35% (5). Effective RA management necessitates early diagnosis, a treat-to-target approach, and the attainment of remission or low disease activity (6). Achieving optimal therapeutic success remains the main challenge in RA, as only 16 % of patients reach sustained remission or low disease activity (7, 8). This has been particularly underscored by the recent recommendations from the European Alliance of Associations for Rheumatology (EULAR), particularly concerning the management of difficult-to-treat patients with RA (9).

The understanding of cellular and molecular mechanisms underlying disease activity has garnered substantial attention. Notably, specific cell subsets, such as synovial tissue macrophages, have been associated with both remission and disease activity (10). Additionally, several synovial molecular and pathobiological markers have shown promise in predicting treatment response (11–13). The emergence of bulk transcriptomic data has further revealed that alterations in synovial and blood transcriptomic profiles were closely associated with disease activity and flares (14–16). In the pursuit of comprehensive insights, single-cell RNA-Seq (scRNA-Seq) emerges as a powerful tool to simultaneously profile cell subset compositions and cell type–specific transcriptional states, enabling a deeper understanding of mechanisms associated with nonremission. Several studies have utilized single-cell resolution to investigate RA, although the majority of research has focused on synovial tissue and none specifically studied disease activity (17–19).

Recent cross-tissue metaanalyses of transcriptome data, encompassing samples from both human and murine models, have uncovered genes associated with disease activity and have underscored a divergence between synovium and peripheral blood profiles (20, 21). Consequently, it is crucial to recognize that markers identified in the synovium cannot be directly extrapolated to those found in peripheral blood. The accessibility of peripheral blood, compared with the more invasive nature of synovial sampling, emphasizes its practical advantage for both research and potential clinical applications. An additional challenge in studying RA disease activity is the inherent heterogeneity of the condition and the potential influence of demographic factors, such as sex, age, ethnicity, and race, on disease activity (22–25). A critical aspect of addressing this challenge involves promoting the establishment of more standardized and diverse cohorts, enabling a better exploration of specific cell subsets and biomarkers that contribute to disease activity.

In this study, we describe a comprehensive analysis of disease activity using scRNA-Seq of peripheral blood mononuclear cells (PBMC) in a diverse cohort of patients with RA, matched with controls based on age, sex, race, and ethnicity. Our primary objective was to identify specific cell subsets and biomarkers associated with disease activity. Additionally, we aimed to assess the specific RA cell subsets and gene signatures in a diverse population, providing valuable insights into the multifaceted nature of RA pathogenesis.

## Results

### Experimental study design.

**Figure 1:**
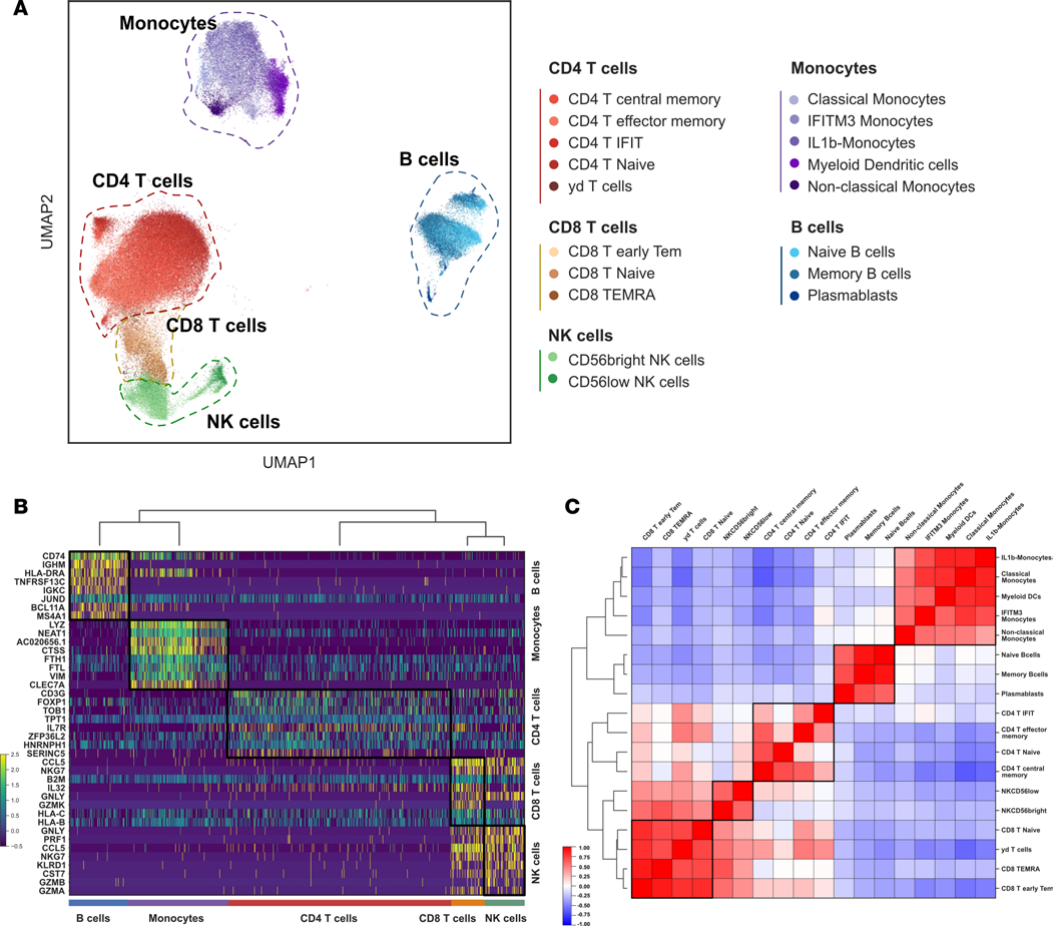
Identification of 18 PBMCs cell subsets. (**A**) UMAP embeddings and subset annotations of scRNA-Seq data set from patients with rheumatoid arthritis (*n* = 18) and healthy controls (*n* = 18) matched on age, sex, and ethnicity. (**B**) Normalized expression of the top 40 ranked genes in different cell subsets (Wilcoxon rank test, FDR ≤ 0.05). (**C**) Correlation heatmap of gene expression across cells subsets (Spearman correlation). CD, cluster differentiation; IFIT, IFN-induced proteins with tetratricopeptide repeats; IFITM, IFN-induced transmembrane; Tem, T effector memory; TEMRA, terminally differentiated effector memory; RA, rheumatoid arthritis.

We performed a single-cell analysis on PBMC samples from 36 participants (18 RA and 18 controls matched on age, sex, ethnicity, and race). Study design and population characteristics are described in Figure 1A and Table 1. The mean age was 53.75 ± 15.9 (mean ± SD), the study was composed of 66.7% (*n* = 24) women, 11.1% Asian Americans, 83.3% White, and 5.6% Latinx population. There was no significant difference in population characteristics between patients with RA and matched controls (Supplemental Table 1; supplemental material available online with this article; https://doi.org/10.1172/jci.insight.178499DS1). Among the patients with RA, 62.5% (*n* = 10) presented a positive rheumatoid factor (RF) and 87.5% (*n* = 14) presented positive anti-citrullinated antibodies (ACPA). RA disease activity was evaluated using the Disease Activity Score (DAS) on 28 joints using C-reactive protein (CRP) (DAS28-CRP) (26). Clinical data regarding disease activity were available for 16 of the 18 patients with RA included in our study. The mean DAS28-CRP was 3.3 ± 1.0. Patients were stratified in remission-low disease activity (DAS28-CRP < 3.2) (*n* = 9) and moderate-high disease activity (DAS28-CRP ≥ 3.2) (*n* = 7). Erosive disease was present in 62.5% of patients (*n* = 10). The mean time since diagnosis was 4.13 ± 4.41 years. Seven patients were treated by conventional disease-modifying antirheumatic drugs (DMARDs) (38.9%), only 1 patient was treated by a biological DMARDs (TNF inhibitor: etanercept), and 6 (33.3 %) patients were also treated with oral corticosteroids (prednisone ≥ 5 mg)

### Identification of 18 PBMC cell subtypes.

**Figure 2:**
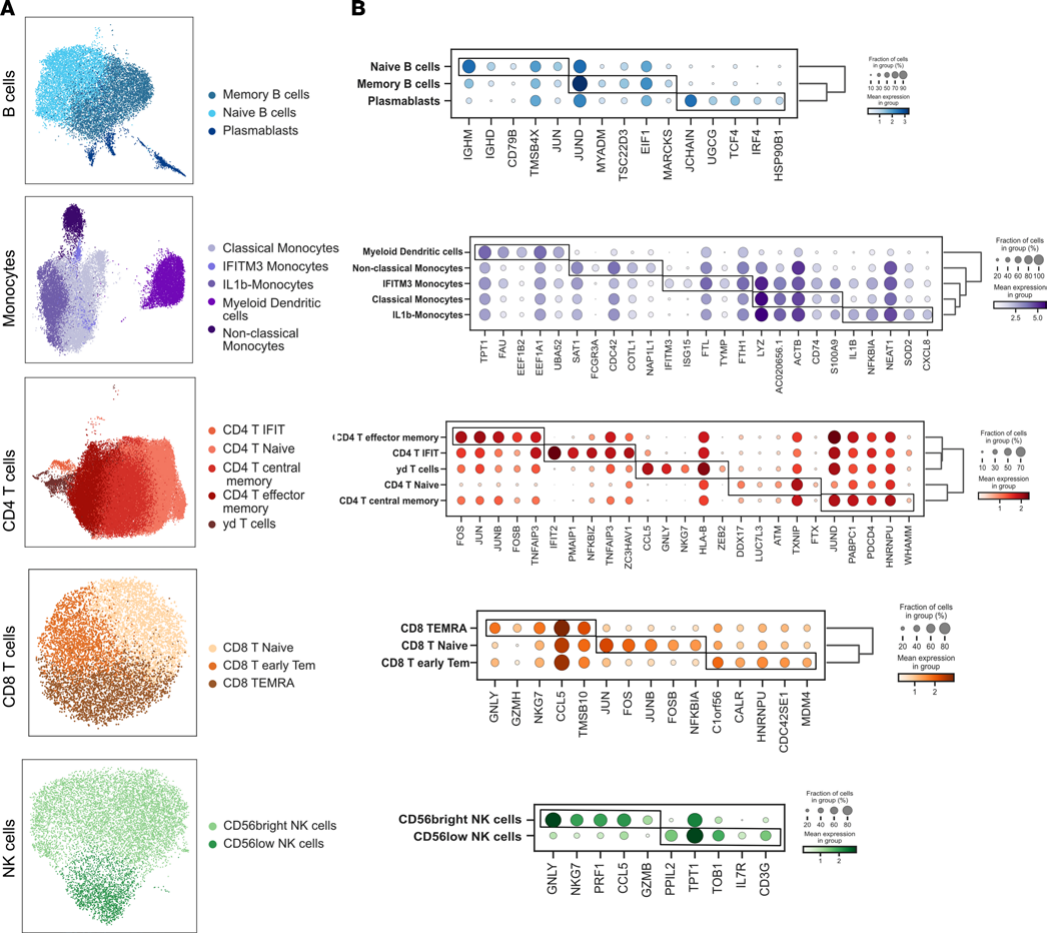
Cell subsets and top marker genes identified in Wilcoxon rank sum. (**A**) UMAP embedding for cell subsets in B cells, monocytes, CD4 T cells, CD8 T cells, and NK cells. (**B**) Dot plots of top ranking genes in each cell subset. CD, cluster differentiation; IFIT, IFN-induced proteins with tetratricopeptide repeats; IFITM, IFN-induced transmembrane; Tem, T effector memory; TEMRA, terminally differentiated effector memory; RA, rheumatoid arthritis.

PBMCs collected on peripheral blood were pooled, profiled, and barcoded in 3 batches with 12 lanes using the 10X Genomics Chromium Single Cell technology. RA and matched controls were evenly split within each batch and within lanes. Cell Ranger v3 was used for demultiplexing and read mapping to the human genome. The mean number of cells for all samples described was 5,990.7 (SD, 1842.8) before filtering and 3,307.8 (SD, 1031.7) afterward. An average of 1,259.2 cells were filtered out per sample as doublets, and an average of 1,423.7 cells per sample were filtered out due to low quality (Supplemental Figure 14 and Supplemental Tables 8 and 9). Following 10X sequencing and preprocessing with Scanpy, our data set consisted of 125,698 cells and 22,159 genes (Supplemental Figure 1 and Supplemental Tables 6 and 7). Leiden community detection was used to group cells into clusters, and annotation using established cell markers showed the presence of all major PBMC cell types (Supplemental Figure 2). All major cell types, annotated using established cell markers, were present in our data set. Further clustering and annotation were used to identify cell subsets, including 5 CD4^+^ T cell subsets (CD4^+^ T central memory, CD4^+^ T effector memory, CD4^+^IFIT^+^ T cells, CD4^+^ naive T cells, γδ T cells), 3 CD8^+^ T cell subsets (CD8^+^ early T effector memory, CD8^+^ naive T cells, terminally differentiated effector memory [TEMRA]), 2 NK cell subsets [CD56^bright^ NK cells,CD56^lo^ NK cells), 3 B cell subsets (naive B cells, memory B cells, and plasmabasts), and 5 monocyte subsets (classical monocytes, IFN-induced transmembrane 3–positive [IFITM3^+^] IFN-activated, IL-1B, myeloid DCs, nonclassical monocytes) (Figure 1B and Supplemental Table 10). For the control sample that was replicated across batches, we found no statistically significant differences in cell proportions. Each of the cell subsets presented a distinct expression profile (Figure 1C and Supplemental Figure 2). CD4^+^ and CD8^+^ T cell and NK cell subtypes showed higher similarity profiles, and the expression profiles of γδ T cells exhibited a stronger correlation within the CD8^+^ T cells (Figure 1C). Cell subsets and top genes of each cell subset identified through Wilcoxon ranked sum analysis are included in Figure 2, A and B. We identified 2 cell subtypes (CD4^+^IFIT^+^ cells and IFITM3^+^ IFN-activated monocytes) associated with genes related to IFN-pathway activation in RA. The proinflammatory CD4^+^IFIT^+^ cell subtype presented significant expression of several genes associated with inflammatory response and immune regulation, including *IFIT2*, *PMAIP1*, *NFKBIZ*, *TNFAIP3*, and *ZC3HAV1*. IFITM3^+^ IFN-activated monocytes had elevated levels of *IFITM3*, *ISG15*, *FTL*, *TYMP*, and *FTH1*. In addition, IFITM3 expression was specific to this monocyte subset (Supplemental Figure 4). Nonclassical monocytes cell proportions were significantly lower in patients with RA compared with controls (Wilcoxon-signed rank analysis *P* = 0.024) (Supplemental Figures 3 and 5). There were no significant differences in the proportion of other cell types.

### Pseudobulk differential expression analysis reveals a specific downregulation of proinflammatory genes related to γδ T cells in patients with RA in comparison with healthy controls.

**Figure 3:**
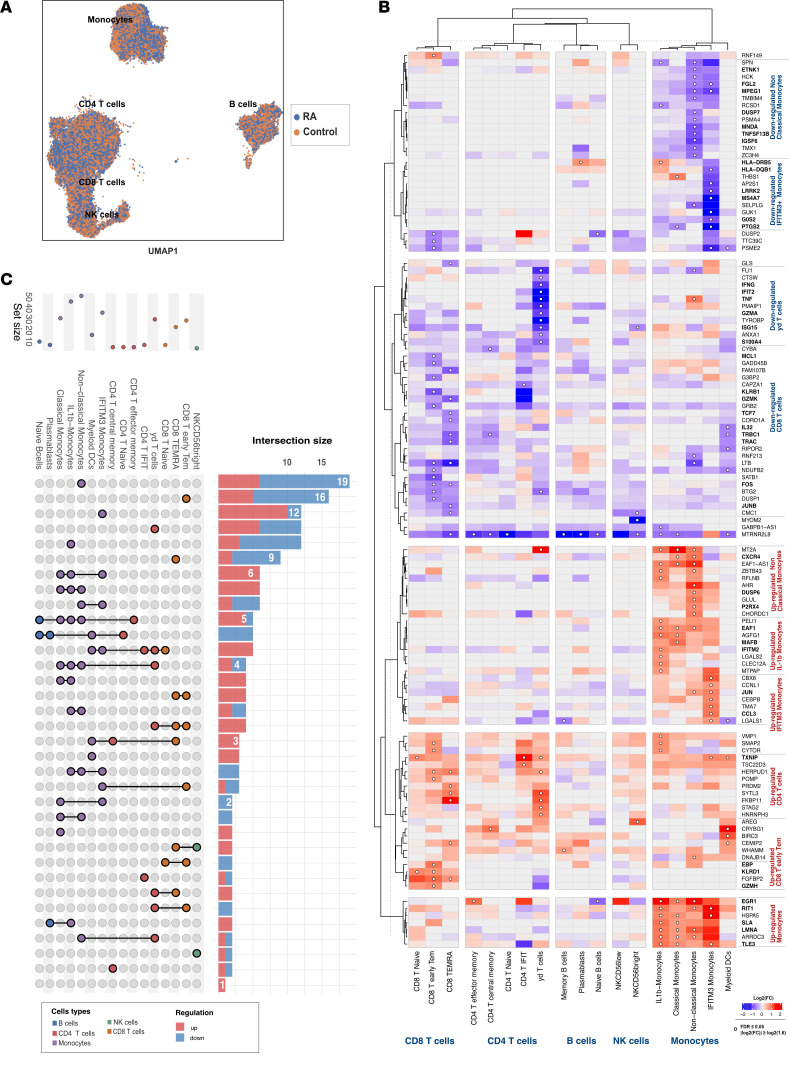
Pseudobulk analysis between patients with RA and matched controls for each subsets. (**A**) Single-cell UMAP of patients with RA and matched controls. (**B**) Differentially expressed genes between patients with RA and matched controls. (**C**) UpSet plots of upregulated and downregulated genes across different cell subsets. CD, cluster differentiation; IFIT, IFN-induced proteins with tetratricopeptide repeats; IFITM, IFN-induced transmembrane; Tem, T effector memory; TEMRA, terminally differentiated effector memory; RA, rheumatoid arthritis.

By performing pseudobulk differential expression analysis on 18 cell subtypes, we identified a total of 168 genes that exhibited differential expression between individuals with RA and the control group (FDR ≤ 0.05, |log_2_FC| ≥ log_2_[1.6]). The majority of those genes were expressed in monocytes (*n* = 94) and CD8^+^ T cells (*n* = 39); 26 genes were expressed in CD4^+^ T cells, 6 were expressed in B cells, and 3 were expressed in NK cells (Figure 3, A–C; Supplemental Tables 2 and 3; and Supplemental Figure 6). In total, 121 genes were unique, and 47 genes were expressed across multiple cell types. Patients with RA had higher expression of genes associated with inflammation and cardiovascular risk in IL-1B and classical monocytes, including *IFITM2*, *TXNIP*, *EAF1*, *RIT1*, *EGR1*, *TLE3*, and *SLA*. In addition, they showed overexpression of cytotoxic genes *KLRD1*, *GZMH*, and *EBP* in CD8^+^ T cells. Patients with RA also displayed significant downregulation of proinflammatory genes such as *IFNG*, *IFIT2*, *TNF*, *GZMA*, *ISG15*, and *S100A4* exclusively in the γδ T cells. Nonclassical monocytes showed a specific transcriptomic profile of 19 differentially expressed genes not shared with other cell subsets (Figure 3, B and C), including a downregulation of *ETNK1*, *TNFSF13B*, *DUSP7*, and *IGSF6* and an upregulation of *CXCR4* in RA. Interestingly, the IFN-activated subset also presented a cell type–specific downregulation of *HLA-DQB1*, *LRRK2*, *MS4A7*, and *G0S2* in RA.

### Overrepresentation analysis finds significant upregulation of B cell activation in patients with RA.

**Figure 4:**
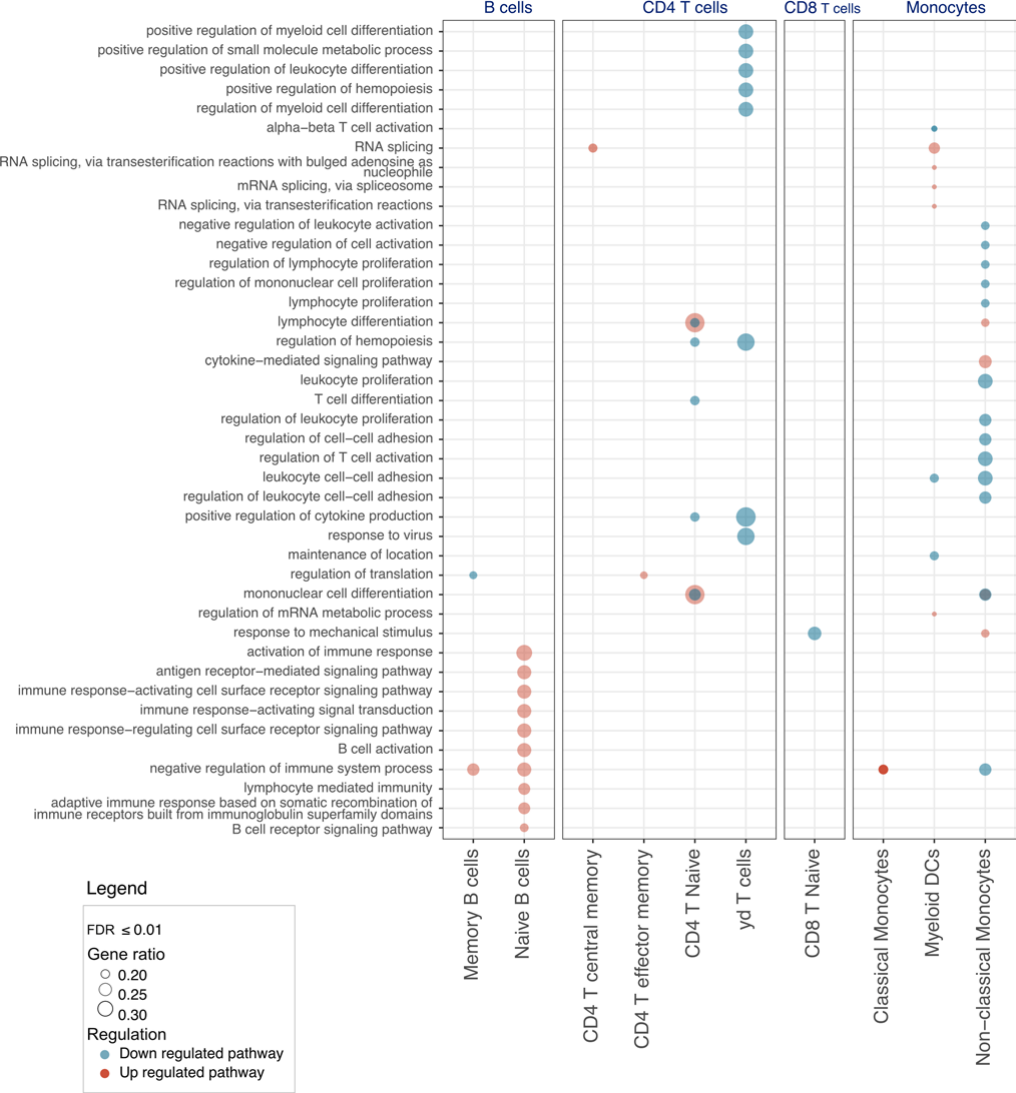
Functional analysis between RA and matched controls. Pathways and overrepresentation analysis for each cell subtype (gene ratio > 0.15, FDR ≤ 0.05, 0.08 < base mean < 4). CD, cluster differentiation; IFIT, IFN-induced proteins with tetratricopeptide repeats; IFITM, IFN-induced transmembrane; Tem, T effector memory; TEMRA, terminally differentiated effector memory; RA, rheumatoid arthritis.

Functional analysis derived from pseudobulk differential expression analysis (FDR ≤ 0.05) identified 25 significantly upregulated pathways across cell subsets in patients with RA compared with the control group. Among these pathways, 11 were upregulated in B cells, 10 in monocytes, and 4 in CD4^+^ T cells. Additionally, 21 pathways were significantly downregulated, with 16 in monocytes, 13 in CD4^+^ T cells, 1 in CD8^+^ T cells, and 1 in B cells (FDR ≤ 0.01, gene ratio ≥ 15, counts ≥ 5) (Figure 4 and Supplemental Table 4). We observed a noteworthy abundance of upregulated pathways specifically in B cells of patients with RA as compared with the control group. These pathways primarily encompassed immune response, B cell activation, B cell receptor pathways, antigen-receptor–mediated signaling pathways, and immune response regulating cell surface receptor signaling pathways. Furthermore, within the γδ T cell population, we observed a significant downregulation of pathways involved in positive regulation of myeloid and leukocyte differentiation as well as cytokine production regulation in patients with RA. Finally, in nonclassical monocytes, we observed an upregulation of cytokine-mediated signaling pathways and a downregulation of pathways involved in T cell activation, lymphocyte regulation and mononuclear cell proliferation, and leukocyte cell-cell adhesion in patients with RA.

### CD4^+^ central memory cell and nonclassical monocytes proportions are associated with disease activity in patients with RA.

**Figure 5:**
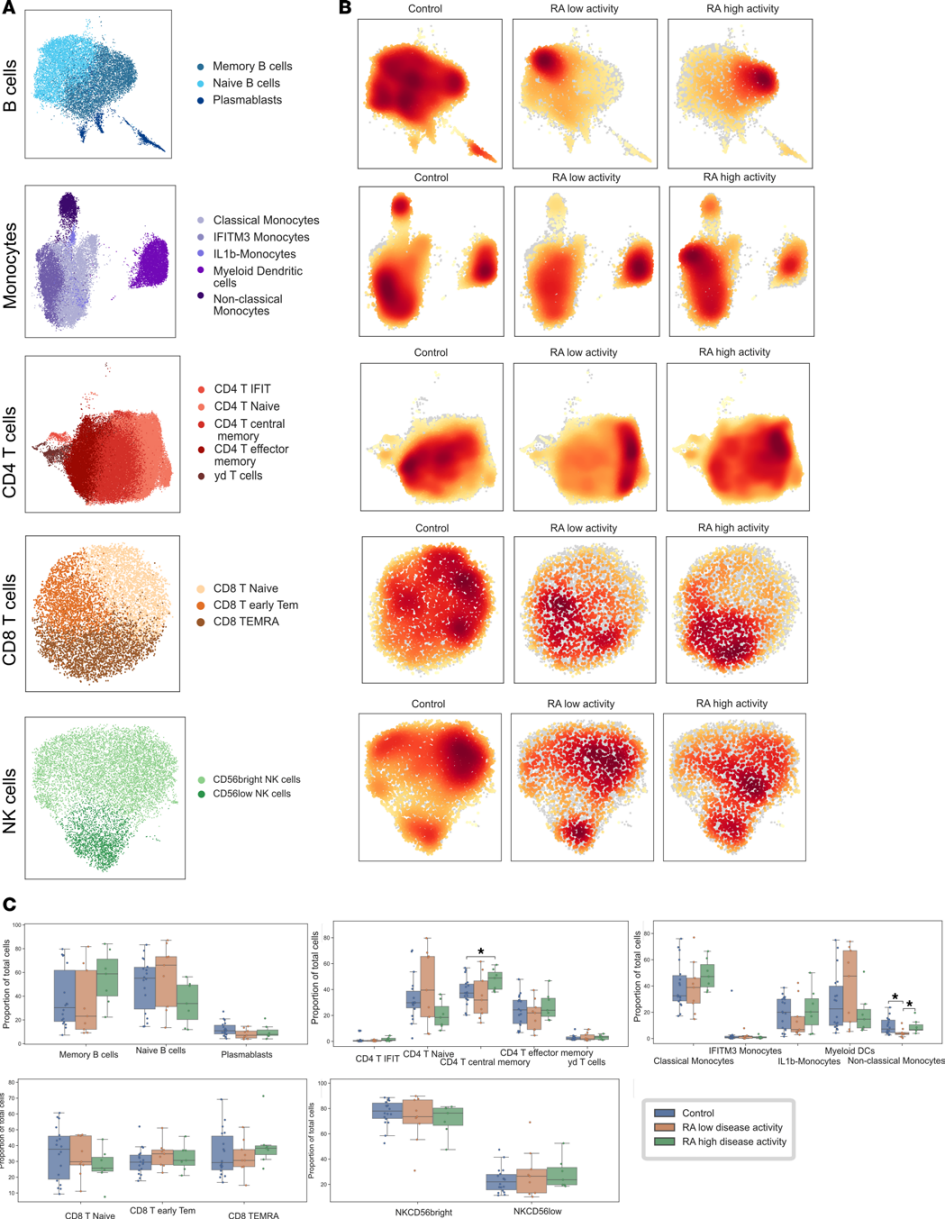
Cell proportion and cell density between patients with remission-low and moderate-high disease activity and matched controls. (**A**) UMAP representation of cell subsets. (**B**) Compositional and density analysis between control patients with low and high disease activity. (**C**) Cell proportion analysis between controls and patients with RA with ow and high disease activity. Each point represents the cell subset proportion of each patient normalized to the total number of cells for that patient (Mann-Whitney *U* test, *P ≤ 0.05). CD, cluster differentiation; IFIT, IFN-induced proteins with tetratricopeptide repeats; IFITM, IFN-induced transmembrane; Tem, T effector memory; TEMRA, terminally differentiated effector memory; RA, rheumatoid arthritis.

We performed a stratified analysis based on disease activity comparing individuals with active versus inactive disease. Information regarding DAS4-28-CRP was available in 16 of 18 patients with RA included in the study. Patients with RA were divided into 2 groups: one consisting of individuals in remission or with low disease activity, characterized by a DAS28-CRP <3.2 (*n* = 9), and the other group composed of patients with moderate and high disease activity, indicated by DAS28-CRP ≥ 3.2 (*n* = 7). There was no significant statistical difference between the 2 groups in terms of age, sex, race, ethnicity, BMI, proportions of ACPA or RF, erosive disease, treatment strategy, or disease duration (Table 1). We compared differences in cell density and cell subset proportions between those 2 groups and the control group (Figure 5, A–C). Additionally, we conducted a nonparametric Spearman correlation analysis to evaluate the association between cell proportions and DAS28-CRP as a continuous score (Supplemental Figure 7). Although there was no statistical significance in B cell proportion across groups, we observed a clear density shift between patients with remission-low disease activity versus moderate and high activity from naive to activated memory B cells (Figure 5B). Patients with RA with moderate-high disease activity showed a significantly increased proportion of CD4^+^ central memory cells (*P* = 0.034) (Figure 5C). Conversely, nonclassical monocytes were significantly lower in patients in the remission-low disease activity group compared with both the control group and the group with moderate-high disease activity (*P* = 0.022).

### Identification of a gene signature specific to moderate and high disease activity in RA.

**Figure 6:**
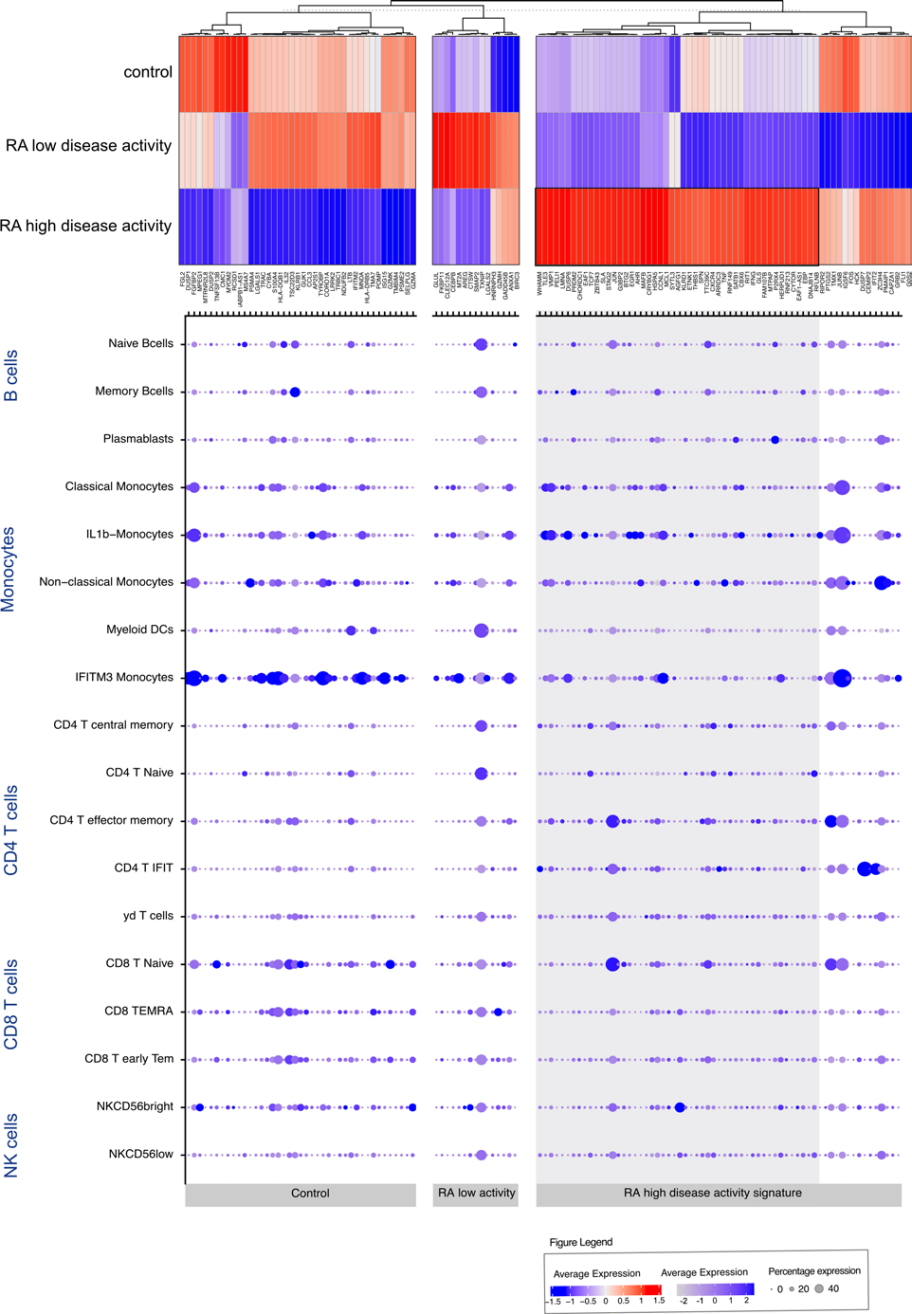
Gene signature associated with disease activity and percentage of expression across cell subsets. Gene expression heatmap between controls and RA with low and high disease activity and average expression across cell subtypes. CD, cluster differentiation; IFIT, IFN-induced proteins with tetratricopeptide repeats; IFITM, IFN-induced transmembrane; Tem, T effector memory; TEMRA, terminally differentiated effector memory; RA, rheumatoid arthritis.

Using a gene list consisting of 121 unique genes that exhibited differential expression between individuals with RA and matched controls in our pseudobulk analysis, we conducted a subanalysis focusing on patients with different disease activity levels: those in remission or with low disease activity and those with moderate-high disease activity. Among the 121 genes, 75 were significantly associated with low disease activity, and 89 were associated with high disease activity (FDR ≤ 0.05, |log_2_[FC]| ≥ log_2_[1.6], 0.08 ≤ base, mean < 4). Interestingly, 52 genes were significantly upregulated in patients within the moderate-high disease activity group. These genes included *G0S2*, *THBS1*, *DUSP7*, *IFIT2*, *IGSF6*, *MAFB*, *RIT1*, *TNF*, *JUN*, *CXCR4*, and *TLE3* (Figure 6 and Supplemental Figure 8). Furthermore, we observed a separate set of 37 genes that exhibited a significant downregulation in patients with moderate-high disease activity compared with the control group. These downregulated genes included *TRBC1*, *KLRB1*, *IL32*, *HLA-DQB1*, *HLA-DRB5*, *TNFSF13B*, *CCL3*, *LRRK2*, and *TMA*7. Additionally, we identified 12 genes that were significantly overexpressed only in patients with remission or low disease activity; they included *TXNIP*, *LGALS2*, and *AREG*.

### CD4^+^ T cells and B cell subsets are associated with the highest levels of cell-cell communication in patients with RA.

**Figure 7:**
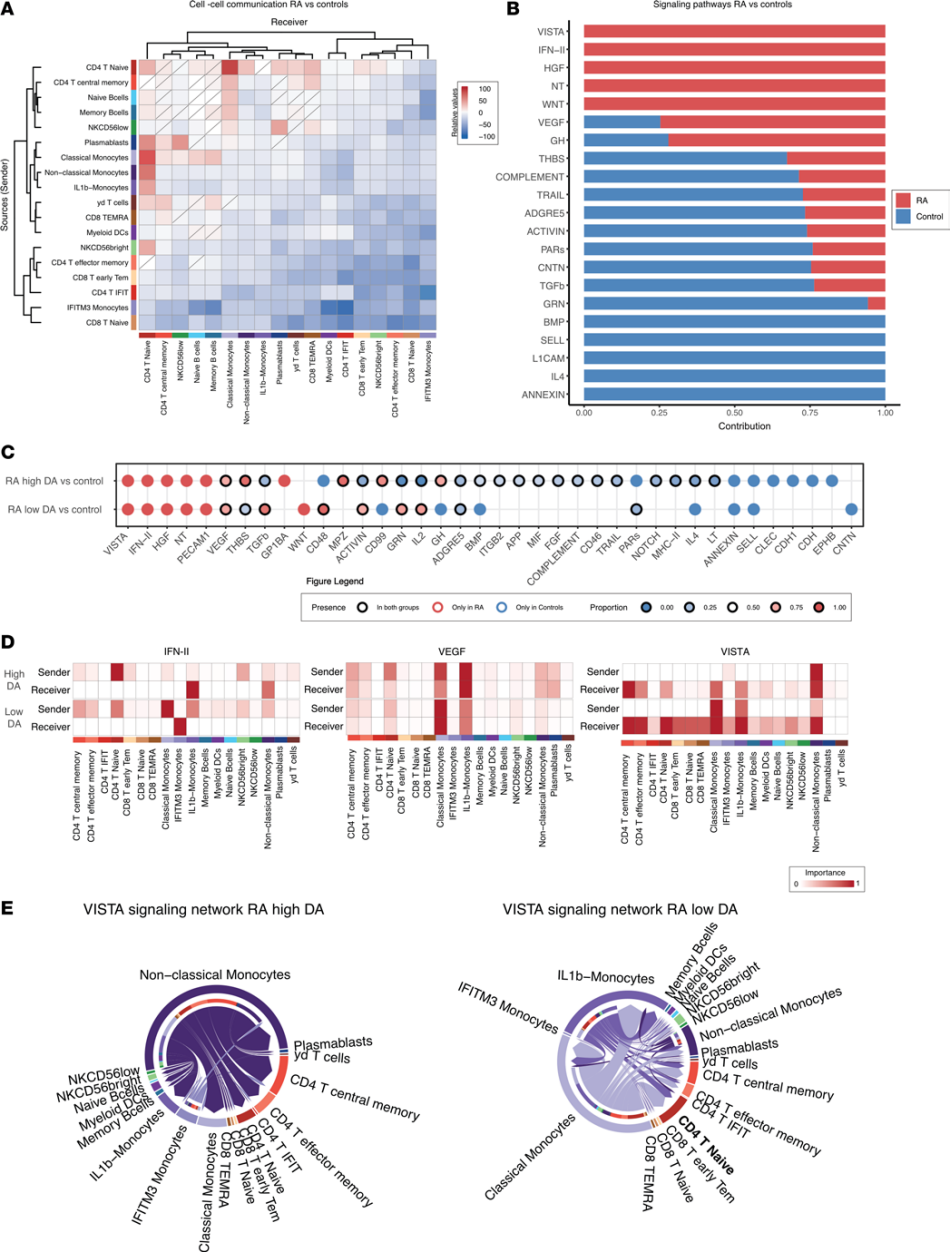
Cell-cell communications between patients with remission-low and moderate-high disease activity and matched controls. (**A**) Heatmap representing the relative number of interactions between RA and matched controls. (**B**) Bar plot illustrates statistically significant communication pathways based on the weight of interactions between patients with RA and controls. (**C**) Dot plot of the relative contribution of communication pathways based on weight of interactions between high and low disease activity compared with controls. (**D**) Heatmaps of the relative importance of cells as senders and receivers for the IFN-II, the VEGF, and the NT signaling pathway network in high and low disease activity. (**E**) Circle plots representing the relative importance of cells as senders and receivers for the VISTA signaling pathway network in high and low disease activity and overall. CD, cluster differentiation; IFIT, IFN-induced proteins with tetratricopeptide repeats; IFITM, IFN-induced transmembrane; Tem, T effector memory; TEMRA, terminally differentiated effector memory; RA, rheumatoid arthritis.

To gain a comprehensive understanding of immune cell communication, we conducted a cell-cell communication inference analysis using CellChat, which uses a repeated permutation to identify significant cell-cell communications. We found a statistically significant increase in cell-cell communication in patients with RA as compared with healthy controls in 35 pairs of cell types. One cell-cell pair showed no difference, and 255 pairs had less communication in RA (Figure 7A). The largest increase in communication was found in CD4^+^ naive T and CD4^+^ T central memory cells along with naive and memory B cells both as senders and receivers. In addition, there was an increase between classical, nonclassical, and IL-1β monocytes as senders and naive CD4^+^ T cells as receivers. When stratifying patients based on disease activity, we found similar patterns. In patients with low disease activity, 28 pairs had statistically significant increases and 286 pairs had statistically significantly decreased in communication as compared with controls (Supplemental Figure 9). In patients with moderate and high disease activity, 37 pairs had statistically significant increases in communication and 259 pairs presented with statistically significant decreases in communication, while 1 pair had a similar amount of communication as compared with controls. For the high activity states, a decrease as both sender and receiver was observed in particular for CD8^+^ T naive and early T effector memory (TEM) and CD4^+^ TEM as well as CD56^bright^ NK cells and IFITM3 IFN-activated monocytes, whereas the low disease activity state had a decrease of both sending and receiving communication of IFIT^+^CD4^+^ T cells, myeloid DCs, and IFITM3 IFN-activated monocytes. On the other hand, myeloid DCs appeared to be more involved in communication in high disease activity as both a sender and receiver, while NK cells and CD4^+^ naive T cells were more involved in sending and receiving communication in low disease activity.

### Cell-cell communication reveals an upregulation of VISTA and IFN-II pathways in patients with RA with moderate and high disease activity.

CellChat utilizes a curated database of ligand-receptor pairs, grouped into communication pathways that may contain multiple ligand-receptor pairs. The change in communication pathways between disease states was then calculated as the relative contribution per disease state to the total communication amount for a specific communication pathway. We used a threshold for significant contribution at less than 35% or more than 65% of the total communication and *P* < 0.05. We found several distinct communication pathways to be up- or downregulated in RA, with 7 pathways showing more communication and 14 showing less communication as compared with controls (Figure 7B and Supplemental Figure 10). Upregulated pathways included neurotrophic (NT), hepatocyte growth factor (HGF), V-domain immunoglobulin suppressor of T cell activation (VISTA), interferon II (IFN-II), and WNT. When comparing moderate-high disease activity to low disease activity, we found the highest number of changed pathways to be in moderate-high (36 versus 22) (Figure 7C and Supplemental Figure 11, A–C). Many pathways from the initial comparison remained significantly and solely expressed in RA, including NT, HGF, VISTA, and IFN-II, although PECAM-1 also became significant. For each pathway, specific ligand-receptor pairs were considered main contributors (Supplemental Figure 12).

For most pathways, the direction of dysregulation was similar in both high and low disease activity (*n* = 12). However, the thrombospondin (THBS), CD99, and growth hormone (GH) pathways were upregulated in high disease activity but downregulated in low disease activity. On the other hand, there was more communication in TGF-β, activin, granulin (GRN), and IL-2 pathways in low disease activity as compared with high disease activity. Although the direction of dysregulation was alike for high and low disease activity, the cells contributing to communication pathways differed. For IFN-II, naive CD4^+^ T cells, and CD56^bright^ NK cells sent signals to primarily IL-1B and nonclassical monocytes in high disease activity (Figure 7D). In low disease activity, most signaling came from classical and IL-1B monocytes and was received by IFITM3 IFN-activated monocytes (Figure 7D). In other pathways, such as VEGF and NT, the involved cells remained unchanged across disease states; however, small changes in importance were observed (Figure 7, C and D, and Supplemental Figure 13). For IL-2, most communication was between CD4^+^ cells, although myeloid DCs also played a large role in high disease activity (Supplemental Figure 13). Another upregulated pathway across disease states was the VISTA pathway, for which we found discrepancies in the involved cells, as nonclassical and IFITM3 IFN-activated monocytes were the primary senders in high disease activity as opposed to low disease activity, during which nonclassical monocytes were out shadowed by classical and IL-1B monocytes (Figure 7E). For both disease states, however, the main receivers were within the CD4^+^ and myeloid subsets, although CD4^+^ naive T cells were more prominent receivers in low disease activity and CD4^+^ memory T cells were more prominent in high disease activity.

## Discussion

Here we describe a data set of scRNA-Seq of PBMCs from a diverse population of 36 patients with 18 RA and 18 matched controls on age, sex, race, and ethnicity. Of these subsets, we found that nonclassical monocyte proportions were significantly lower in patients with RA compared with controls. This is in line with other studies of RA biomarkers, which hypothesize that inflammatory cell subsets may migrate from blood to synovial tissue (20, 21, 26). Our findings showing increased CD4^+^ central memory cell proportions and decreased nonclassical monocytes specifically in patients with moderate-high disease activity compared with controls provide further evidence aligned with these hypotheses. However, unlike previous studies (27), we did not find statistically significant enrichment of plasmablasts or other cell subsets in RA patient populations, which may be due to low sample sizes or differing patient demographics from previous studies. One other subset of interest identified in our study was an IFITM3-expressing IFN-activated monocyte subset. IFITM3 is associated with type I IFN response and viral restriction (28). Associations between IFITM3 haplotypes and RA, particularly in the context of a Korean population (29). Additionally, Zhang and colleagues (17) identified upregulation of IFITM3 in synovial IL-1B^+^ monocytes from a subset of patients in RA, prompting interest in whether differences in IFITM3^+^ cell proportions could be contributing to these differences in expression. Other IFN-induced genes, particularly *OAS1*, *ISG15*, *IFI44L*, and *IFI6* (30), have also been shown to be upregulated in whole blood samples from patients with RA, although these studies did not find any association with disease activity. Studies on IFN-activated monocytes have also suggested their role in antigen presentation through upregulation of CD86 and HLA-DR and driving the differentiation of Th17 cells in the synovium of patients with RA (31).

While we did not find differences in IFITM3^+^ monocyte proportions or upregulation of the IFITM3^+^ gene specifically, our other findings described below did show significant differences in VISTA signaling in this subset and showed upregulation of IFN-induced genes in pseudobulk analysis from patients exhibiting moderate and high disease activity. The differences between our study and previous findings from Zhang and colleagues may be explained by varying study design, including cohort selection and control populations (17). While we compared PBMC samples between patients with RA stratified by disease activity and healthy controls, upregulation of IFITM3 shown by Zhang and colleagues was only shown in synovial samples from leukocyte-rich RA compared with control samples obtained from patients with osteoarthritis. Further studies of IFITM3 and other IFN-related genes are needed to better elucidate the global role of IFN and IFN-activated monocytes in RA pathophysiology.

In our pseudobulk differential expression analysis, we observed a specific downregulation of proinflammatory genes in the γδ T cells subsets, including *IFNG*, *IFIT2*, *TNF*, *GZMA*, *ISG15*, *S100A4*. Interestingly Mo et al. described that peripheral Vδ2T cells were significantly lower in patients with RA and were negatively correlated with disease activity. In addition, they described that Vδ2 T cells from RA accumulated in the synovium and produced high levels of proinflammatory cytokines including IFN-γ and IL-17 and also showed elevated chemotaxis potential (32). Our results could reinforce the potential chemotaxis role of Vδ2 T cells in the synovium. However, it is essential to acknowledge that this remains a hypothesis, since we lacked matched tissue data in our study to confirm this hypothesis conclusively. Similarly, we observed a lower proportion of nonclassical monocytes in PBMCs from patients within the remission-low disease activity groups compared with both controls and moderate-high disease activity. Guła et al. have reported that the absolute number of circulating nonclassical monocytes negatively correlates with DAS28 and swollen joint count in patients with peripheral spondyloarthritis (26). Nonclassical monocytes have also been associated as key mediators of tissue destruction in osteoclasts in murine models of RA (33). In addition, we found a specific downregulation in the nonclassical monocytes subsets of several genes such as *IGFS6*, *ETNK1*, *DUSP7*, and *TNFSF13B*. *IGFS6* expression has been significantly associated with RA fibroblast cells in humans (34). *Etnk1* has been associated as a candidate gene in collagen-induced arthritis (35). *DUSP7* is involved in MAPK signaling, and low levels of mRNA of *DUSP7* have been associated with RF ACPA^+^ patients with RA (36). *TNFSF13B* variants have been associated with RA in several studies and also with other autoimmune diseases such as systemic erythematous lupus (37, 38).

We also identified a gene signature of 89 genes specific to disease activity, including an upregulation of proinflammatory genes such as *TNF*, *JUN*, *EGR1*, *IFIT2*, *IGSF6*, *TMX1*, and *MAFB* as well as potential genes associated as therapeutic targets or in treatment response such as *G0S2*, *PTGS2*, and *THBS1*. *Mafb* has been associated with monocytes and macrophage differentiation but also involved in the activation of myeloid cells associated with joint destruction such as RANK^+^TLR2^–^ cells in murine models of RA (38, 39). *G0S2* has been associated with anti-TNF response prediction in a metaanalysis of 11 studies (40). Thrombospondin-1 expression has been associated with NR4A2 activity and is modulated by TNF inhibitors (41). We also observed several genes downregulated in patients with high disease activity such as *HLA-DQB1*, *HLA-DRB5*, and *TNFSF13B*. Similarly, Klimenta et al. also found a protective role of HLA-DRB5 in RA (42). In an independent single cell study, Wu et al. showed that HLA-DRB5^+^ expression was lower in the synovial tissues of ACPA^–^ patients with RA (18). A thorough analysis of these gene signatures enables the identification of various functional groups, notably the upregulation of IFN-induced genes in patients exhibiting moderate to high disease activity. These findings align with existing literature that emphasizes the significant role of IFN in both RA and disease activity (31, 43). Furthermore, they underscore the potential of cutting-edge RA treatments, such as JAK inhibitors, as IFN exerts its effects through the JAK/STAT pathway (44).

Cell-cell communication allowed us to confirm several well-known signaling pathways from the RA literature, including type II IFN (IFN-γ), TGF-β, and VEGF (43, 45, 46). Additionally, our findings reveal an upregulation of the IL-2 signaling pathway in patients with remission and low disease activity, whereas a downregulation was observed in patients with moderate and high disease activity compared with controls. IL-2 has been correlated with disease activity and severity in several studies (47, 48). The IL-2 pathway plays a pivotal role in Treg response and holds significance in rheumatic diseases (49). IL-7 is known to play a significant role in the activation and proliferation of many cells, and notably T cells, including Tregs (50).

IL-7 is also important for the development and differentiation of Tregs, their homeostatic maintenance, and their expansion, although to a lesser extent than IL-2, which is indispensable (51, 52). In addition, the IL-7R shares the common γ chain and, thus, part of the downstream activation pathway. Our results underscore the intricate interplay between these cytokines and their downstream signaling cascades and may signify that the shared IL-2/IL-7 pathways and genes are particularly relevant in the context of RA pathogenesis. However, it should be acknowledged that our study lacked sufficient power to identify specific memory and Treg subsets and did not identify IL-2 or IL-7 in our differential expression gene list. Thus, further research is warranted to refine and enhance this hypothesis.

We also found an upregulation of the VISTA signaling pathway in patients with RA. VISTA is a negative checkpoint regulator, playing a key role in suppressing T cell–mediated immune responses, and its disruption has been linked to proinflammatory phenotypes and a susceptibility to autoimmune diseases (53). In a recent groundbreaking study, ElTanbouly et al. investigated the role of VISTA expression in T cells and found that VISTA expressed on naive T cells was playing a critical role in quiescence and peripheral tolerance and, hence, that blocking or knocking out VISTA exacerbates inflammation in mice models (54). However, results from Ceeraz et al. have found a significant reduction of arthritis score in VISTA-deficient mice in a collagen antibody–induced arthritis model independent of T and B cells (55). Taken together, those results could suggest that VISTA may have immunoregulatory roles on naive T cells but inflammatory roles on myeloid cells. In our signaling network analysis, however, CD4^+^ T cells subsets were the main receivers of VISTA signaling. CD4^+^ naive T cells were the primary receiver population in the remission-low disease activity group, while CD4^+^ T central memory was the predominant group in moderate-high disease activity. Interestingly, the monocyte cell population was associated as the main sender in the VISTA signaling network — in particular, IL-1β monocytes and classical monocytes in remission-low disease activity and nonclassical and IFITM3^+^ monocytes in moderate and high disease activity groups. Targeting VISTA shows promising potential as an innovative immunoregulatory therapy (56, 57). Our findings further support the involvement of VISTA in RA and its potential effect on the communication between monocytes and CD4^+^ T cells, possibly indicating a more significant role of VISTA on monocytes as compared with T cells in RA; however, this finding needs confirmation in laboratory studies. Additional research is necessary to gain a comprehensive understanding of VISTA’s role in RA and its apparent multifaceted role in autoimmunity.

Several limitations should be acknowledged in our study. First, it is challenging to use scRNA-Seq of unsorted PBMCs to study very small cell subsets, such as B cells subsets or Tregs, which may have led to their role in disease activity being underestimated and not extensively explored. Additionally, the relatively small sample size of our study may have limited the statistical power to detect subtle differences. This limitation also restricted our ability to thoroughly explore potential differences associated with sex, race, and ethnicity, emphasizing the need for more inclusive representation in future investigations to ensure a comprehensive understanding of RA across diverse populations.

Finally, we acknowledge that RA treatments could be expected to modify the phenotype of PBMCs and that the small sample size of this study doesn’t allow us to explore these differences analytically. Future studies should be designed specifically to explore the effects of treatments on cell type–specific expression at a single-cell level. Prospective single-cell study and longitudinal data on flare and treatment response could also provide further insight, could reinforce previous findings from the work form Orange et al., and could deepen our understanding of RA disease activity (15). Another limitation is the absence of matched disease synovial tissue, which could have provided more comprehensive insights into how patterns in PBMCs relate to cellular and molecular mechanisms involved in RA synovium. Also, we have looked strictly at PBMCs, but a significant role of neutrophils in RA has been proposed and future studies should explore this potential interaction (58).

Nonetheless, our study provides valuable insights into the cellular and molecular mechanisms associated with disease activity in RA. We carefully considered matched controls on age, sex, race, and ethnicity, and our work has identified key cell subsets and genes that may be associated with disease activity. These findings have the potential to serve as new biomarkers and therapeutic targets. We are optimistic that our research will contribute to advancing our understanding of RA pathogenesis and lead to the development of more effective treatments for this complex autoimmune disease.

## Methods

### Study design.

Patients with RA meeting the American College of Rheumatology (ACR) classification criteria (59) were recruited from the UCSF rheumatology clinic between 2016 and 2020 (60). Healthy controls were recruited through local advertising and through the database ResearchMatch (61). Controls were matched to patients with RA by age, sex, race, and ethnicity. The uses of the words “women” and “men” in this study are utilized as proxies for biological sex and do not represent gender identity. Blood samples and participant level data were collected at time of enrollment. Clinical data including demographics, medication status, and laboratory values, such as erythrocyte sedimentation rate, CRP, RF, ACPA, and clinical measurements of disease activity with the Disease Activity Score in 28 joints using CRP (DAS28-CRP). Patients were stratified in remission or low disease activity (DAS28-CRP < 3.2) and moderate and high disease activity (DAS28-CRP ≥ 3.2) according to the 2019 updated ACR recommendation on disease activity measures (62).

### Sample processing and 10X scRNA-Seq.

PBMCs were isolated from peripheral blood samples by UCSF Bay Area Center for AIDS Research Specimen Processing and Banking Subcore (previously AIDS specimen bank). Blood samples were collected in EDTA tubes, processed per manufacturer’s guidelines, and cryopreserved in liquid nitrogen. scRNA-Seq was performed using the 10X Chromium microfluidics system (10X Genomic). PBMCs from 18 patients with RA and 18 healthy controls were thawed, counted, pooled, and profiled in 3 batches and 12 lanes, using 10X Genomics Chromium Single Cell 3′V3. Barcoded cDNA libraries were prepared using the single cell 3′mRNA kit. Cell Ranger v3 (3.1.0) was used to demultiplex cellular barcodes and map reads to the human (GRCh38-3.0.0) genome (63). Sample deconvolution and doublet identification was performed using demuxlet (64). RA and matched control samples were evenly split within each batch to limit technical and biological bias in our analysis. The first batch consisted of 14 individuals (7 RA and 7 controls), the second batch included 8 individuals (3 RA and 5 controls), and the third batch included 16 individuals (8 RA and 8 controls). One control was sequenced across 3 batches as a technical replicate to control for batch effect.

### Preprocessing.

Preprocessing was performed using Scanpy (1.9.1) (65) following previously published single-cell workflows (66). Additional details on these methods can be found in Supplemental Figures 1–3 and Supplemental Tables 6–9. Genes found in fewer than 3 cells were filtered out, as well as cells containing fewer than 100 genes or more than 1,000 genes. Cells with platelet or megakaryocyte gene markers (*PF4*, *GNG11*, *PPBP*, *SDPR*) were also removed. Additionally, cells containing greater than 20% mitochondrial genes or less than 3% ribosomal genes were removed. Following filtering, the data were normalized to counts per million and were log transformed. Technical variation from sequencing depth, mitochondrial percentage, and ribosomal percentage were regressed out during scaling. Cell cycle scoring was performed using Scanpy using standard genes (67) and was also regressed out. Batch correction was then performed using HarmonyPy (68), and samples were clustered in an unsupervised manner using leiden clustering with a resolution of 3.0. Each cluster was assigned as CD4^+^ T cells, CD8^+^ T cells, monocytes, NK cells, or B cells using manual annotation with predefined marker genes according to the human protein atlas. Clusters of platelets, erythrocytes, and suspected doublets were removed from further analysis based on the presence of marker genes. Subclustering was repeated for each cell type to allow for fine annotations of cell subsets, again based on referenced marker genes and leiden clustering. No size cutoff was used to remove small clusters. Highly expressed genes within each subset were identified using Wilcoxon rank testing implemented in Scanpy.

### Compositional analysis.

Cell densities in each subset were calculated and plotted for RA samples versus controls using Scanpy embedding density functions. The proportion of each cell type within a sample relative to the total number of annotated cells for that sample was also calculated. Cell proportions were compared using Wilcoxon signed rank tests between RA and their matched controls. Additionally, Mann-Whitney *U* tests were used for cell proportions comparison among controls, patients with RA with remission-low disease activity, and patients with RA with moderate-high disease activity. Correlation between cell type proportion and DAS28-CRP was also performed by calculating Spearman rank-order coefficient. Two-sided *P* < 0.05 was the threshold for statistical significance.

### Differential gene expression analysis.

Differential gene expression analysis was performed between RA and matched controls using a pseudobulk approach using the bulk RNA-Seq tool DESeq2 (1.38.3) (69). Pseudobulk methods outperform mixed models and limit pseudoreplication bias (70, 71). For each cell subtype, read counts were summed across each sample to create a pseudobulk count matrix. DESeq2 was applied, using a likelihood ratio test corrected on batch effect with an additional fit of a Gamma-Poisson generalized linear model (GLM) (72). *P* values were adjusted using Benjamini-Hochberg method, and genes with a FDR ≤ 0.05 were selected. Additional filtering was applied with an absolute log_2_fold-change ≥ log_2_(1.6) and a base mean expression between 0.08 and 4.

### Overrepresentation analysis.

Functional and overrepresentation analysis was performed on differentially expressed genes for each cell subtype using clusterprofiler (4.6.2) (73) and Gene Ontology (GO) database (74). We selected up- and downregulated pathways related to biological processes, with a gene ratio ≥ 0.15, count ≥ 5, and FDR ≤ 0.01.

### Cell-cell communication analysis using CellChat.

Cell-cell communication inference and visualization was performed using the CellChat R package (version 1.6.0) (75). CellChat uses the log-normalized expression matrix as input and predicts cell-cell communication based on ligand-receptor pairs in a curated database. For each pair of ligands and receptors, the communication probability is calculated based on the average expression of the ligand in one cell type and the average expression of the receptor in another cell type, taking into account the law of mass action. CellChat also considers other important signaling factors such as heteromeric complexes and cell type proportion in the estimation of the strength of interactions. We followed the standardized CellChat workflow, including the “projectData” function, which allows for projecting the gene expression onto a validated protein-protein interaction network to impute the data. Cell-cell pairwise communication was visualized as the relative number of communications between groups of interest (RA versus control, low disease activity versus controls, and high disease activity versus controls). Statistical significance (FDR-adjusted *P* ≤ 0.05) of cell type sender and receivers was assessed by performing 50 permutations and comparing the results using a Student’s 2-tailed *t* test. Only pathways that were statistically significant (*P* ≤ 0.05) and with a relative contribution in the RankNet-function of either more than 0.65 or less than 0.35 were considered.

### Statistics.

Comparisons of clinical data between groups were performed using 2-tailed Student’s *t* test for continuous variables and χ^2^ test for categorical variables. Differences in cell proportions based on disease activity were assessed using the nonparametric Mann-Whitney *U* test. Correlations between variables were evaluated using Spearman’s rank correlation coefficient. Differential gene expression according to disease activity state was analyzed using the Mann-Whitney *U* test. For differential gene expression, functional, and cell-cell communication analyses, *P* values were adjusted using the FDR with the Benjamini-Hochberg correction. A *P* value of less than 0.05 was considered statistically significant. All statistical analyses were conducted using Python and R programming languages.

### Study approval.

The study was conducted in accordance with the principles outlined in the Declaration of Helsinki and was granted ethical approval by the Human Research Protection Program and the IRB of UCSF (IRB project no. 15-17175). All participants provided written informed consent.

### Data availability.

Data presented in this study are deposited in the CellxGene Discover resource at https://cellxgene.cziscience.com/collections/e1a9ca56-f2ee-435d-980a-4f49ab7a952b The code used for this analysis is publicly available on GitHub at https://github.com/BMiao10/RASingleCell; commit ID 0543692. Values for all data points in graphs are reported in the Supporting Data Values file.

## Author contributions

AJG and LAC recruited the patients for the study. MCN and EN performed the single-cell experiments. DR and WT performed the alignment and the samples deconvolution. MB and BYM performed the quality controls, the preprocessing, and the cell proportion analyses. MB, BYM, and CW contributed to the cell type annotations. MB performed the pseudobulk analysis and the analysis associated with disease activity. MB and CW performed the functional analysis. CW performed the cell-cell communication analysis. EF and UK peer reviewed and provided essential feedback on the code and the analysis strategy. MMY and MCN provided complementary information on the study metadata. MB, BYM, CW, and MMY wrote the manuscript, and MS revised the manuscript. MS, LAC, and MCN conceptualized the study, and MS was involved in the funding acquisition. MS, MK, and AC were involved in the project administration. YS, GKF, JN, JS, DK, EMF, AJB, AJG, CJY, LAC, and MCN provided critical feedback and edits to the manuscript.

## Supplementary Material

Supplemental data

Supporting data values

## Figures and Tables

**Table 1:**
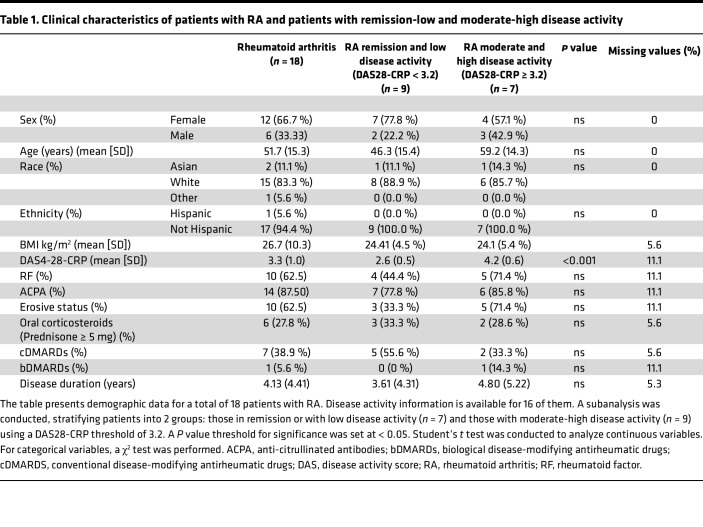
Clinical characteristics of patients with RA and patients with remission-low and moderate-high disease activity

## Version 1 07/02/2024

In-Press Preview

## Version 2 08/22/2024

Electronic publication
