# Supplementary material for: Single-cell RNA-Seq analysis reveals cell subsets and gene signatures associated with rheumatoid arthritis disease activity
Source: JCI Insight. 2024 Jul 2;9(16):e178499. doi: 10.1172/jci.insight.178499 (PMC11343607; doi:10.1172/jci.insight.178499)
Supplement: Supplemental data [file jciinsight-9-178499-s184.pdf]

Conflict of interest: MB's research is funded by a grant from the French Society of Rheumatology, the Osteoarthritis Foundation grant, and a doctoral fellowship from Sorbonne University. BYM is a paid consultant for SandboxAQ. U.K. is a paid consultant for Vevo Therapeutics. DR's research is supported by a grant from Pfizer Aspire, NIH, and the Rheumatology Research Foundation. EF's research is funded by PREMIER and by grants from SICCA and Eli Lilly. GKF's research is funded by grants from NIH, Gerson Baar Foundation, ImmunoX Computational Biology Initiative, Eli Lilly, Bill & Melinda Gates. JS received research grants from Pfizer, MDS, Schwamedico, BMS and received lecture fees from MDS, Pfizer, Abbvie, Fresenius, Kabi, BMS, Roche, Chugai, Sandoz, Lilly, Gilead, Novartis, Janssen. EMF is receiving lecture fees from Sanofi France and expert testimony payment from Takara Bio. AJB's research is funded by NIH and Chan Zuckerberg Initiative grants. AJB is a paid consultant for Maze Therapeutic, HiBio Santa Ana, Trex Bio, ImYoo, DeciBio, and received lecture fees from Boston Children's Hospital, Johns Hopkins University, Endocrine Society, Alliance for Academic Internal Medicine, Roche, Children's Hospital of Philadelphia, University of Pittsburgh Medical Center, Cleveland Clinic, University of Utah, Society of Toxicology, Mayo Clinic, Pfizer, Cerner, Johnson and Johson, the Transplantation society; consultant to Samsung, Mango Tree Corporation, Dartmouth, Gladstone Institute, Boston Children's Hospital; and has received honoraria and travel reimbursement for invited talks from Alliance for Academic Internal Medicine, Cleveland Clinic, University of Utah, Society of Toxicology, Mayo Clinic, Children's Hospital of Philadelphia, American Association of Clinical Chemistry, Analytica, Life Science & Diagnostics Association. AJB receives royalty payments through Stanford University, for several patents and other disclosures licensed to NuMedii, Personalis, and Progenity (Progenity patent number: 11333672). AJB's research has been funded by NIH, Genentech, Johnson and Johnson,Merck,Peraton (as prime for an NIH contract), Priscilla Chan and Mark Zuckerberg Bakar Family foundation.

SUPPLEMENTARY FIGURES:

Figure S1 | Quality control and batch correction with HarmonyPy. A. UMAP projection according to sample type (control or RA) batch and lane before batch correction with Harmony. B. UMAP projection according to sample type, batch and lane after batch correction with Harmony. C. UMAP projection of lanes after batch correction with Harmony. RA, Rheumatoid Arthritis; UMAP, Uniform Manifold Approximation and Projection

Figure S2 | Expression heatmap representing the mean expression of B cells, CD4 T cells, CD8 T cells, NK cells and Monocytes marker genes for cell subsets. DCs, Dendritics cells; Tem, T effector memory; TEMRA, terminally differentiated effector memory

Figure S3 | Cell proportions of B cells, CD4 T cells, CD8 T cells, NK cells and Monocytes across samples. CD, Cluster Differentiation; NK, Natural Killer; RA, Rheumatoid Arthritis

Figure S4 | A. UMAP representation of IFITM3 gene expression across all PBMCs cell types. B. UMAP visualization of IFITM3 gene expression across monocyte subsets. CD: Cluster Differentiation; DCs: Dendritic Cells; IFIT: Interferon Induced proteins with Tetratricopeptide repeats; IFITM: Interferon-induced Transmembrane proteins; Tem: T Effector Memory; TEMRA: Terminally Differentiated Effector Memory. RA: Rheumatoid Arthritis

Figure S5| A. Cell subsets for B cells, Monocytes, CD4 T cells, CD8 T cells, NK cells. B, Compositional analysis, and density plots comparing patients with Rheumatoid Arthritis and matched controls. C. Cell proportion analysis between patients with Rheumatoid Arthritis and Controls (Wilcoxon signed rank test p≤ 0.05). CD, Cluster differentiation; DCs, Dendritic cells; IFIT, Interferon Induced proteins with Tetratricopeptide repeats; IFITM, interferon-induced transmembrane; Tem, T effector memory; TEMRA, Terminally differentiated effector memory; RA, Rheumatoid Arthritis

Figure S6 | Volcano Plot representing the differential gene expression analysis between patients with Rheumatoid Arthritis and matched controls in each cell subset (FDR ≤ 0.05, log2(FC) ≥ log2(1.6), 0.08 ≤ mean expression< 4). CD, Cluster differentiation; DCs, Dendritic cells; FC, Fold change; FDR, False discovery rate; IFIT, Interferon Induced proteins with Tetratricopeptide repeats; IFITM, interferon-induced transmembrane; Tem, T effector memory; TEMRA, Terminally differentiated effector memory; RA, Rheumatoid Arthritis

Figure S7 | A. Spearman correlation of cell subset proportion with the DAS28-CRP for each subset. B Correlation heatmap of cell subset and DAS-28-CRP. CD, Cluster differentiation; DC, Dendritic cells, IFIT, Interferon Induced proteins with Tetratricopeptide repeats; IFITM, interferon-induced transmembrane; Tem, T effector memory; TEMRA, terminally differentiated effector memory.

Figure S8 | Venn Diagram of the genes differentially expressed in RA low and high disease activity compared to controls. (FDR ≤ 0.05, log2(FC) ≥ log2(1.6), 0.08 ≤ mean expression < 4). RA, Rheumatoid Arthritis

Figure S9 | Comparisons of cell-cell communication patterns between patients with low and high disease activity and controls. A. Heatmap representing the relative number of interactions between cell types of RA patients with high disease activity compared to controls. B. Heatmap representing the relative number of interactions between cell types of RA patients with low disease activity compared to controls. DA, Disease activity

Figure S10 | A. Bar plots of all communication pathways based on interaction strength (red are significantly more present in RA, blue are significantly more present in controls). B. Bar plots of all communication pathways based on number. DA, Disease Activity; RA, Rheumatoid Arthritis

Figure S11 | A. Dot-plot of the relative contribution of communication pathways based on number of interactions between high and low disease activity compared to controls. B. Bar plots of all communication pathways based on number. Red are significantly more present in RA (low or high disease activity) and blue are significantly more present in controls. C. Bar plots of all communication pathways based on interaction strength. DA, Disease Activity; RA, Rheumatoid Arthritis

Figure S12 | A. Table of the ligand-receptor pairs that contribute to the communication for the PECAM1, HGF, IFN-II, NT and VISTA pathways. B. Bar plots showing the relative contribution of the ligand-receptor pairs that are involved in the communication for the VEGF and IL2 pathways for high and low disease activity and controls. DA, Disease activity; L, Ligand; R, Receptor.

Figure S13 | Heatmaps of the relative importance of cells as senders and receivers for the VEGF, IL2, HGF, and PECAM1, NT signaling pathway network in high and low disease activity. Controls are also shown for VEGF and IL2. CD, Cluster differentiation; DC, Dendritic cells, IFIT, Interferon Induced proteins with Tetratricopeptide repeats; IFITM, interferon-induced transmembrane; Tem, T effector memory; TEMRA, Terminally differentiated effector memory.

Figure S14 | Quality control metrics used to select filtering criteria. A. Histogram showing distribution of total number of sequencing reads, gene feature counts, and proportions of mitochondrial genes across sequenced cells. B. Violin plots showing the number of total reads and gene feature count identified in each cell, stratified by sequencing lane. C. Scatter plot showing the distribution of cells with high mitochondrial gene proportions, relative to total count and gene feature counts. D. Violin plots showing the proportion of mitochondrial, ribosomal, and hemoglobin associated genes identified in each cell, stratified by sequencing lane.

Figure S1 | Quality control and batch correction with Harmony.py A. UMAP projection according to sample type (control or RA) batch and lane before batch correction with Harmony. B UMAP projection according to sample type, batch and lane after batch correction with Harmony. C UMAP projection of lanes after batch correction with Harmony. RA, Rheumatoid Arthritis; UMAP, Uniform Manifold Approximation and Projection

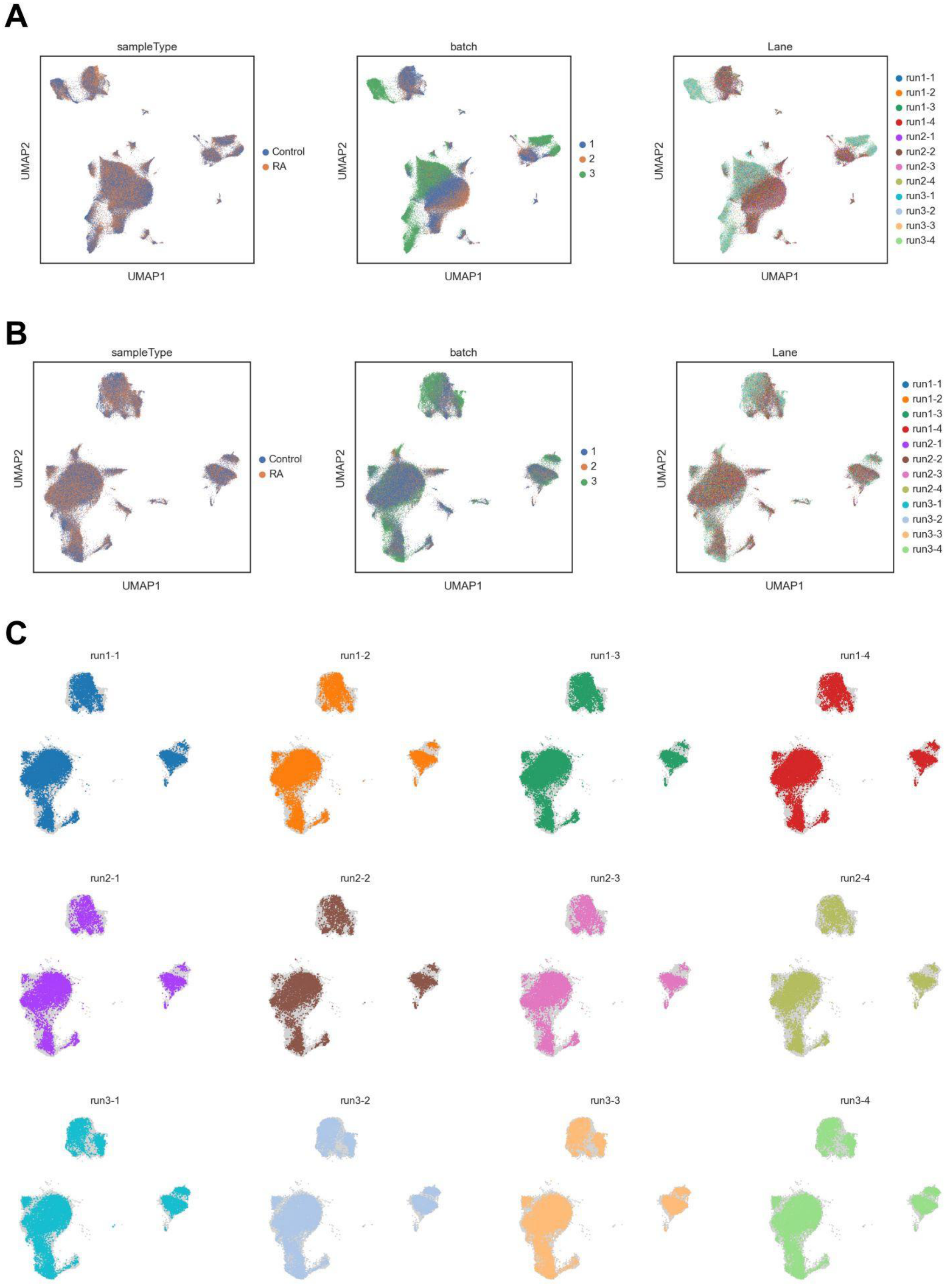

Figure S2 | Expression heatmap representing the mean expression of B cells, CD4 T cells, CD8 T cells, NK cells and Monocytes marker genes for cell subsets. DCs, Dendritic cells; Tem, T effector memory; TEMRA, terminally differentiated effector memory

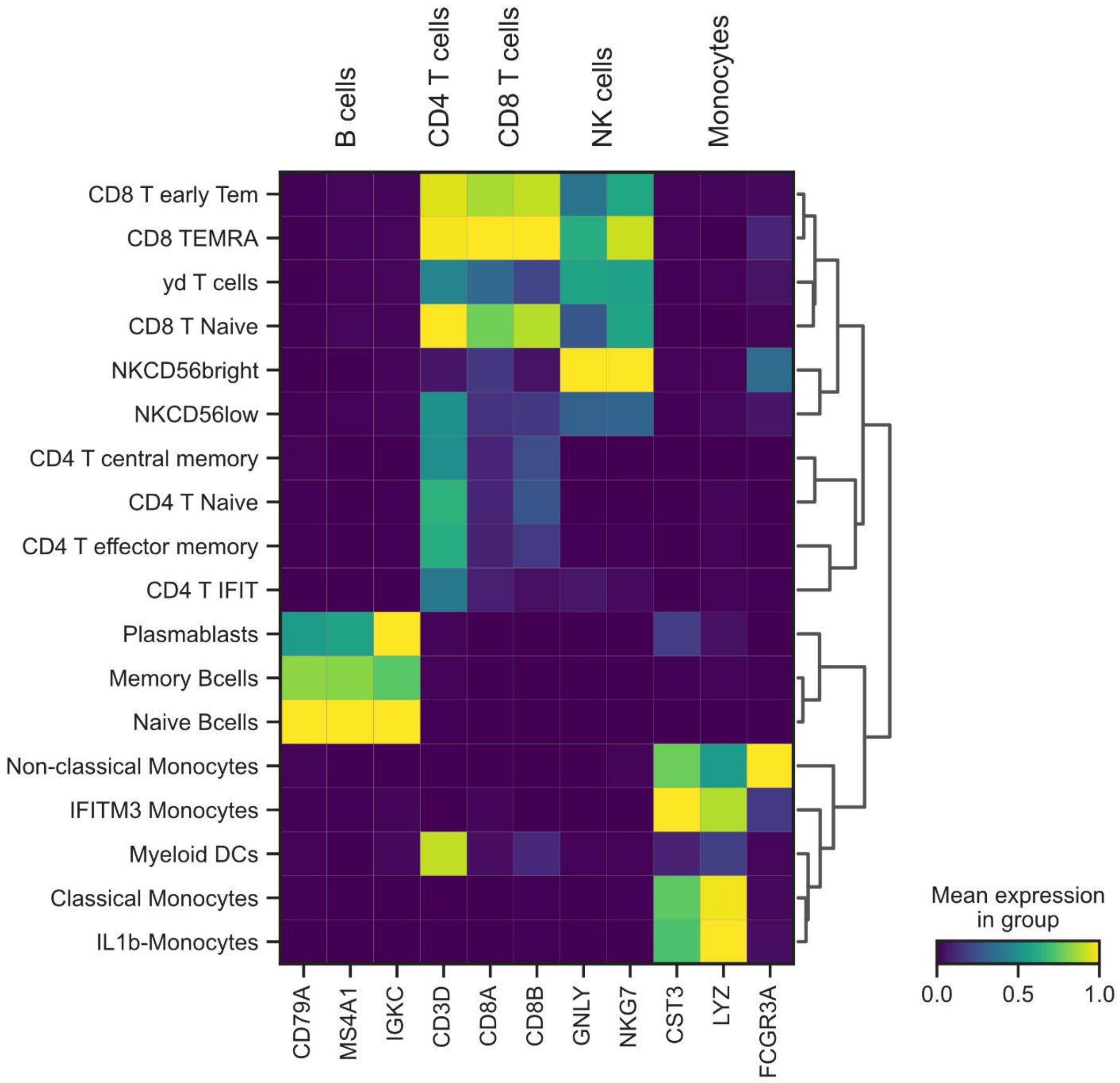

Figure S3 | Cell proportions of B cells, CD4 T cells, CD8 T cells, NK cells and Monocytes across samples. CD, Cluster Differentiation; NK, Natural Killer; RA, Rheumatoid Arthritis

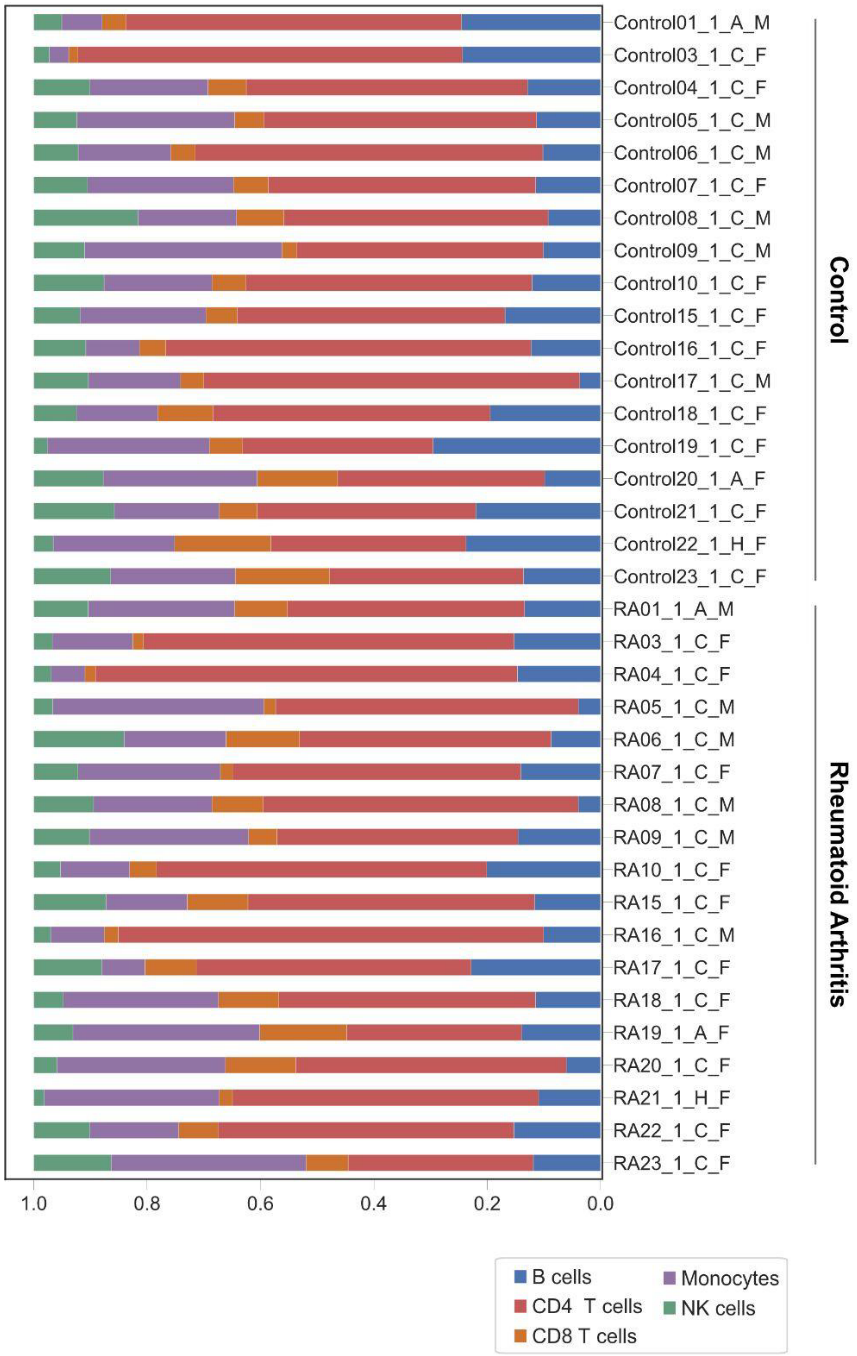

Figure S4 | A. UMAP representation of IFITM3 gene expression across all PBMCs cell types. B. UMAP visualization of IFITM3 gene expression across monocyte subsets. CD: Cluster Differentiation; DCs: Dendritic Cells; IFIT: Interferon Induced proteins with Tetratricopeptide repeats; IFITM: Interferon-induced Transmembrane proteins; Tem: T Effector Memory; TEMRA: Terminally Differentiated Effector Memory. RA: Rheumatoid Arthritis

A

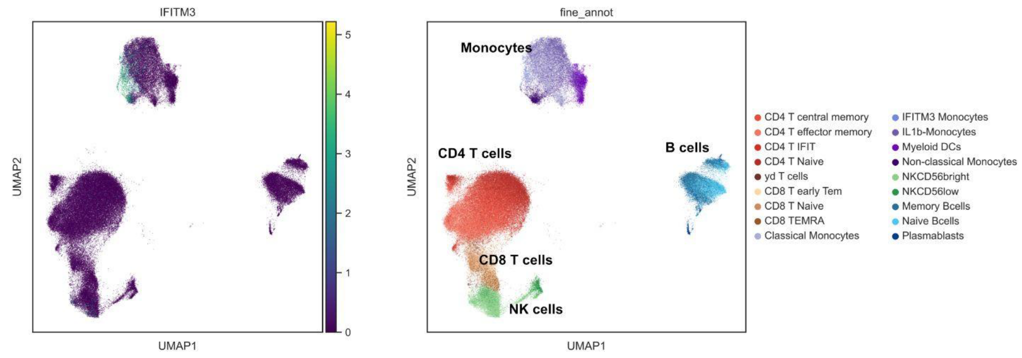

B

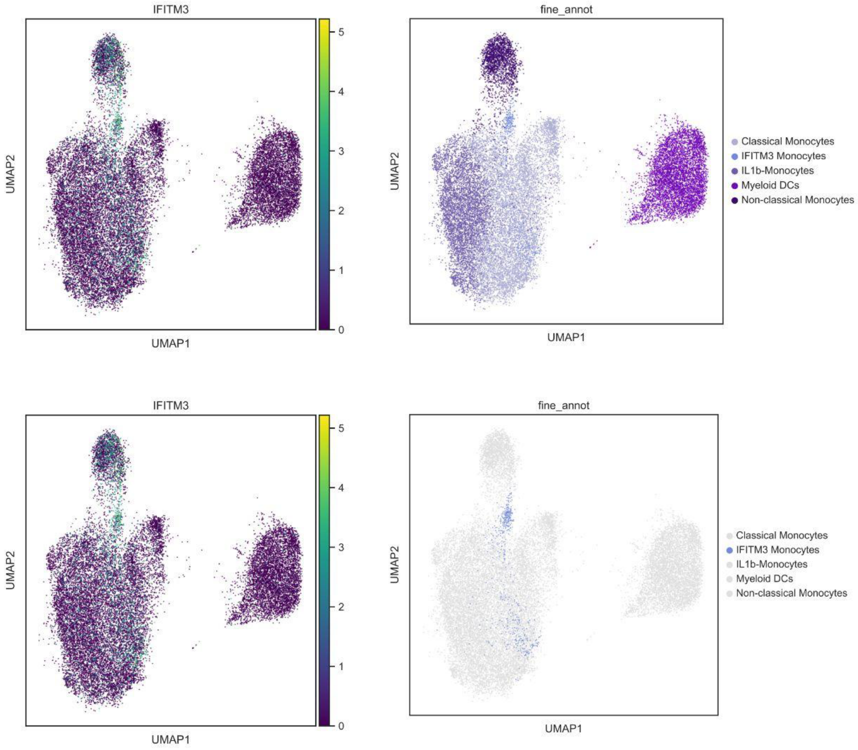

Figure S5 | A. Cell subsets for B cells, Monocytes, CD4 T cells, CD8 T cells, NK cells. B. Compositional and density analysis between patients with Rheumatoid Arthritis and matched controls. C. Cell proportion analysis between patients with Rheumatoid Arthritis and Controls (Mann-Whitney - Wilcoxon p≤ 0.05). CD, Cluster differentiation; DCs, Dendritic cells; IFIT, Interferon Induced proteins with Tetratricopeptide repeats; IFITM, interferon-induced transmembrane; Tem, T effector memory; TEMRA, Terminally differentiated effector memory; RA, Rheumatoid Arthritis

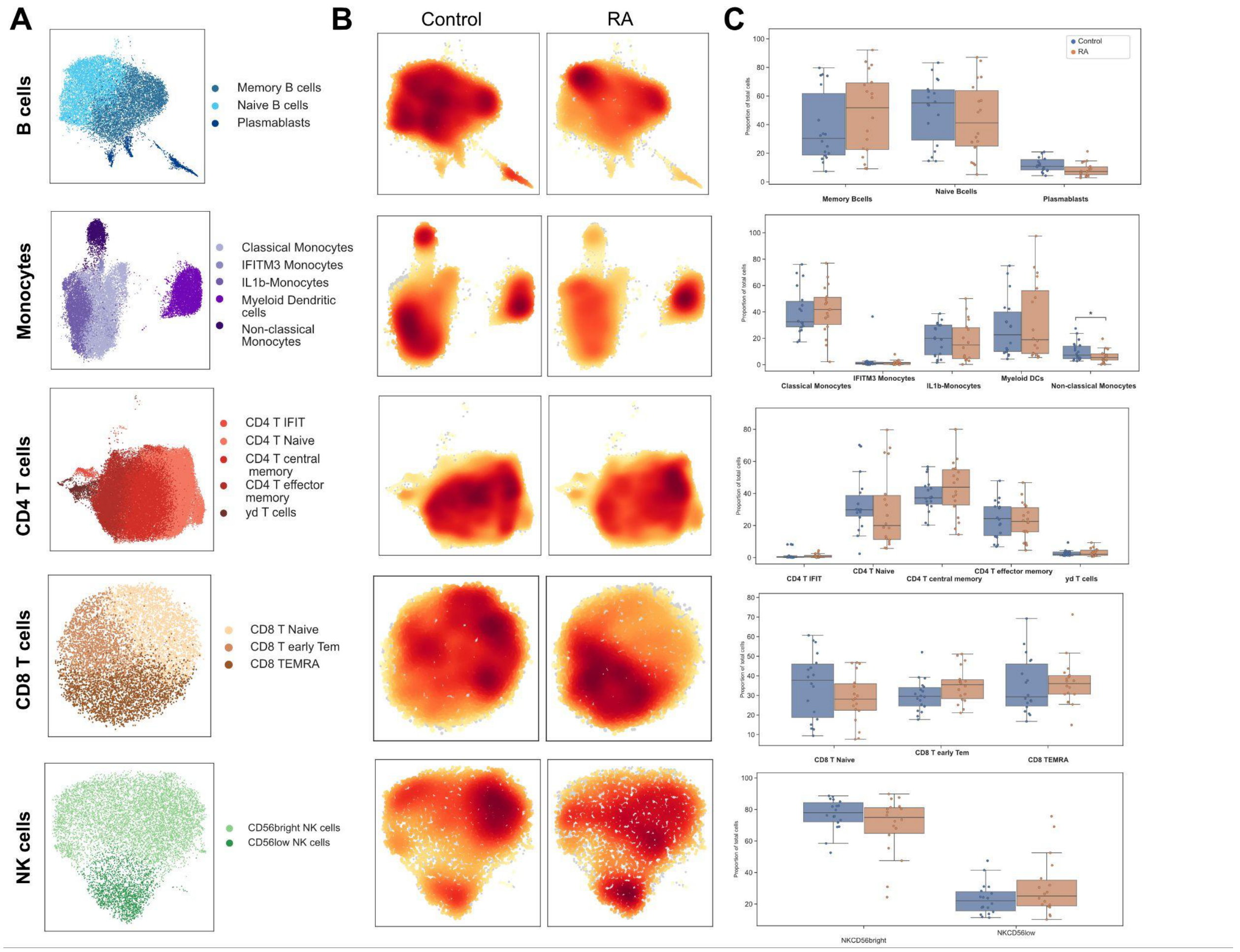

Figure S6 | Volcano Plot representing the differential gene expression analysis between patients with Rheumatoid Arthritis and matched controls in each cell subset (FDR ≤ 0.05, log2(FC) ≥ log2(1.6), 0.08 ≤ mean expression< 4). CD, Cluster differentiation; DCs, Dendritic cells; FC, Fold change; FDR, False discovery rate; IFIT, Interferon Induced proteins with Tetratricopeptide repeats; IFITM, interferon-induced transmembrane; Tem, T effector memory; TEMRA, Terminally differentiated effector memory; RA, Rheumatoid Arthritis

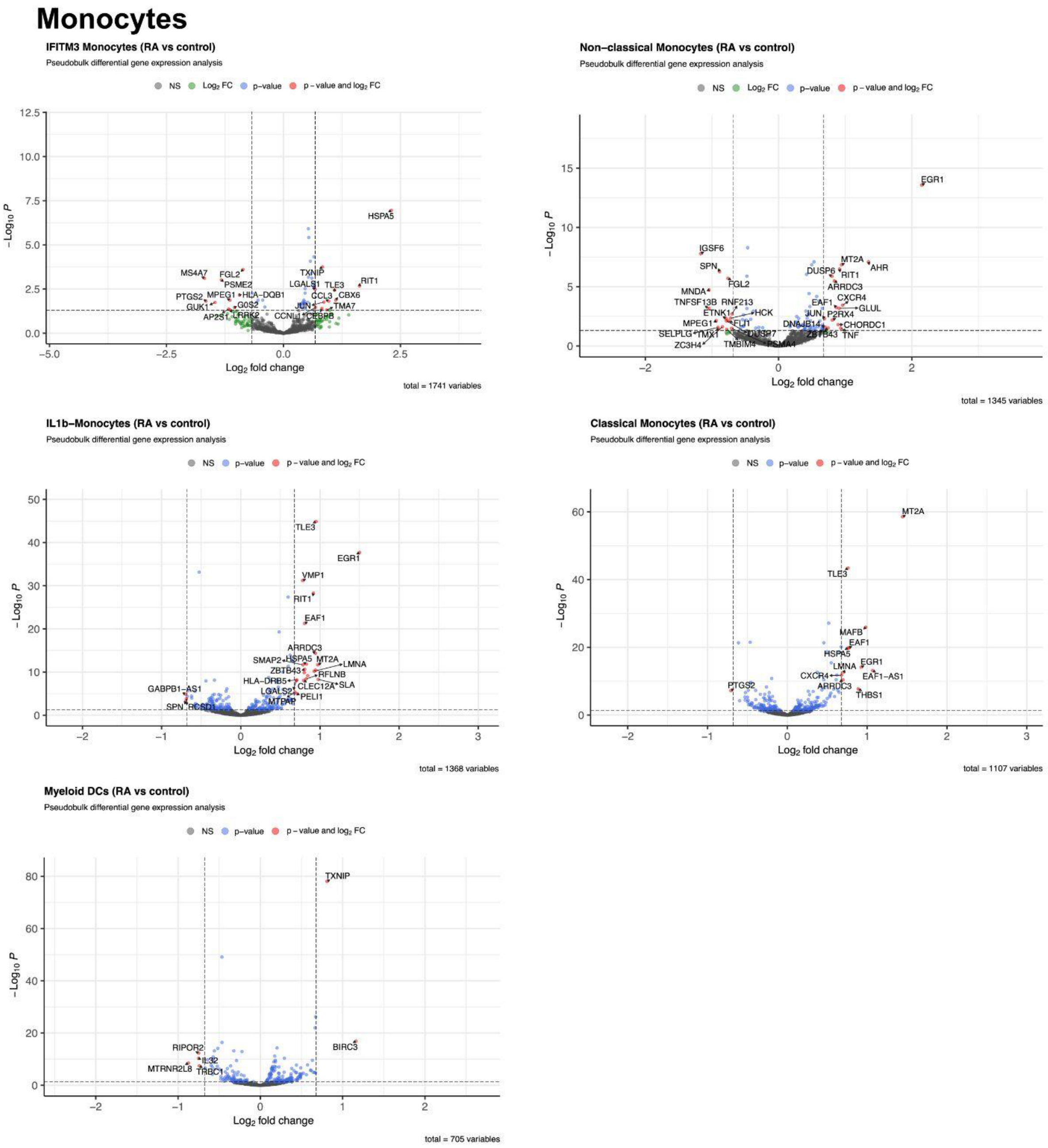

CD4 T cells

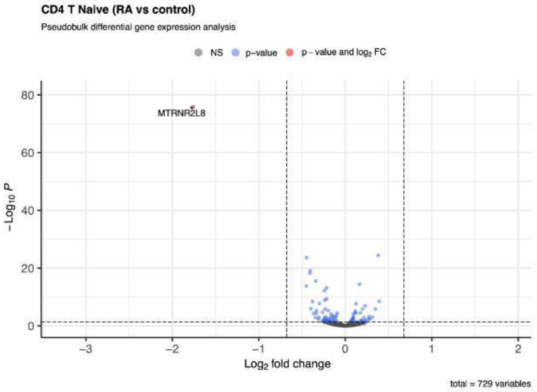

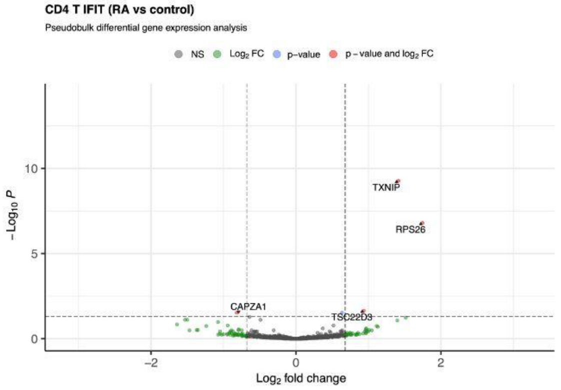

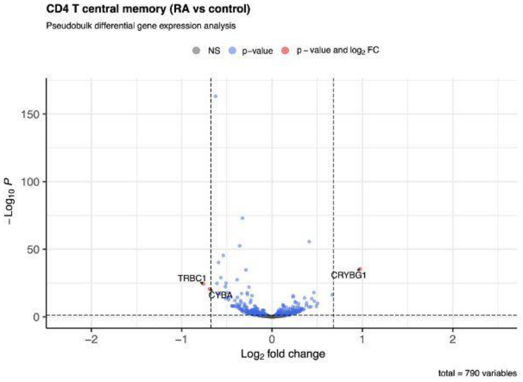

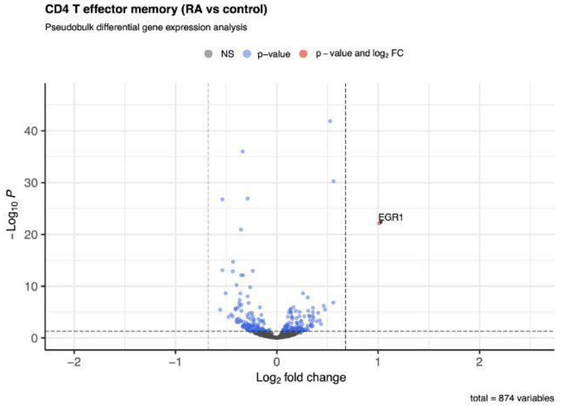

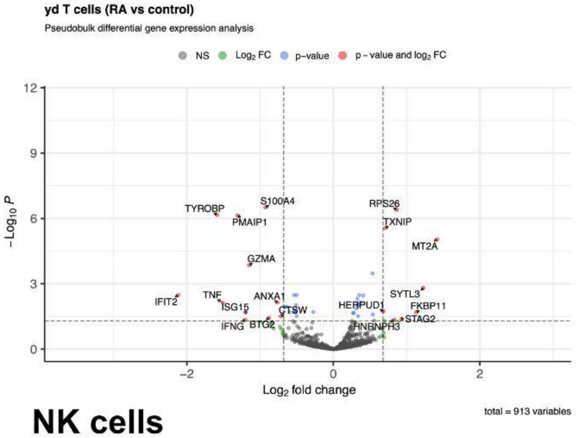

NK cells

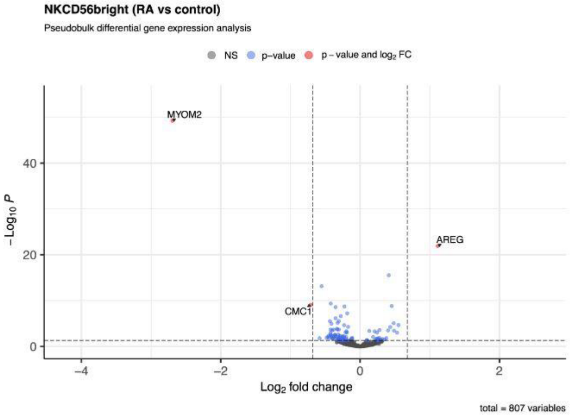

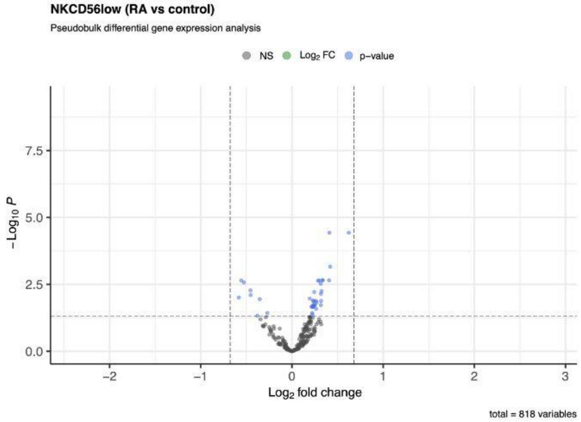

CD8 T cells

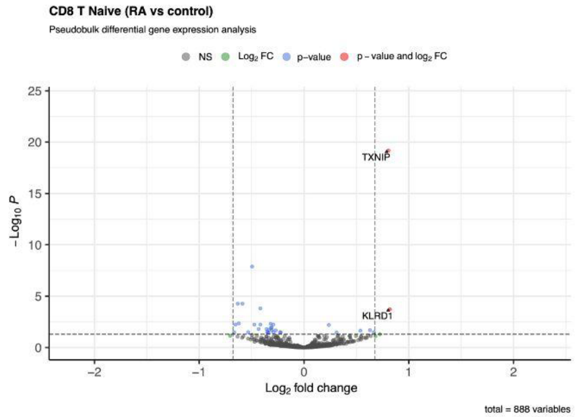

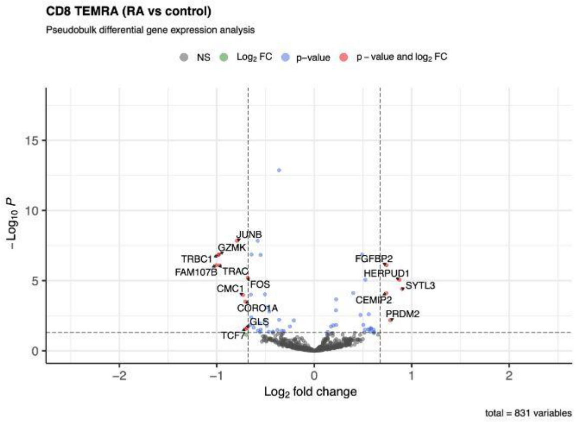

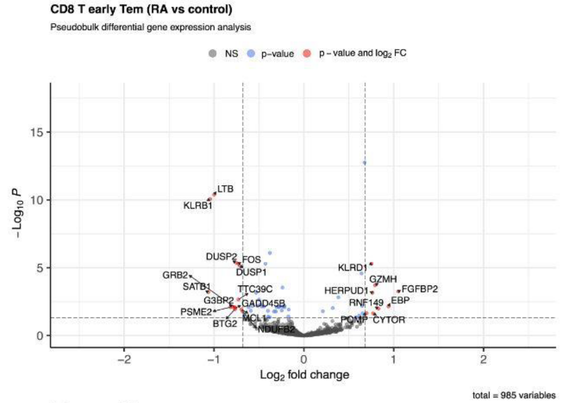

B cells

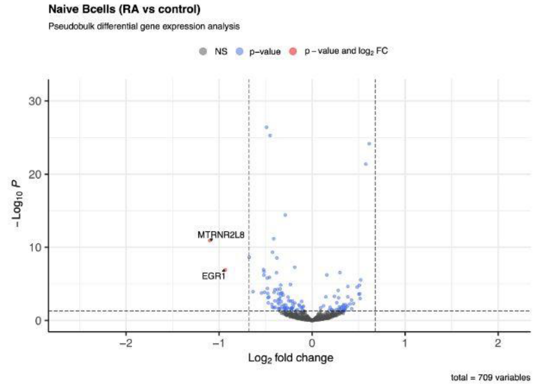

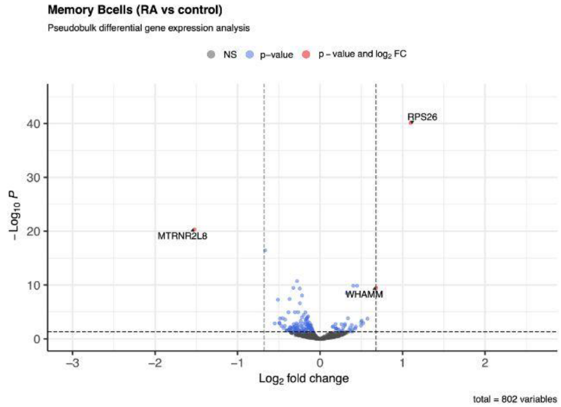

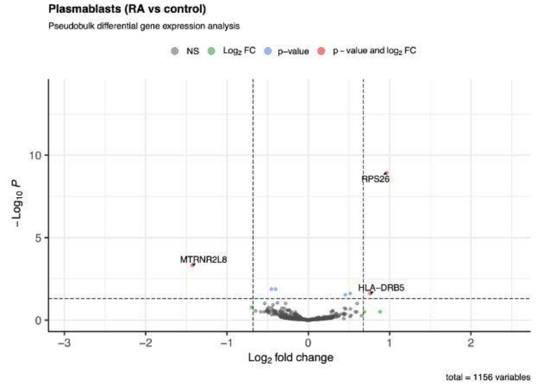

Figure S7 | A. Spearman correlation of cell subset proportion with the DAS28-CRP for each subset. B. Correlation heatmap of cell subset and DAS-28-CRP. CD, Cluster differentiation; DC, Dendritic cells' IFIT, Interferon Induced proteins with Tetratricopeptide repeats; IFITM, interferon-induced transmembrane; Tem, T effector memory; TEMRA, Terminally differentiated effector memory.

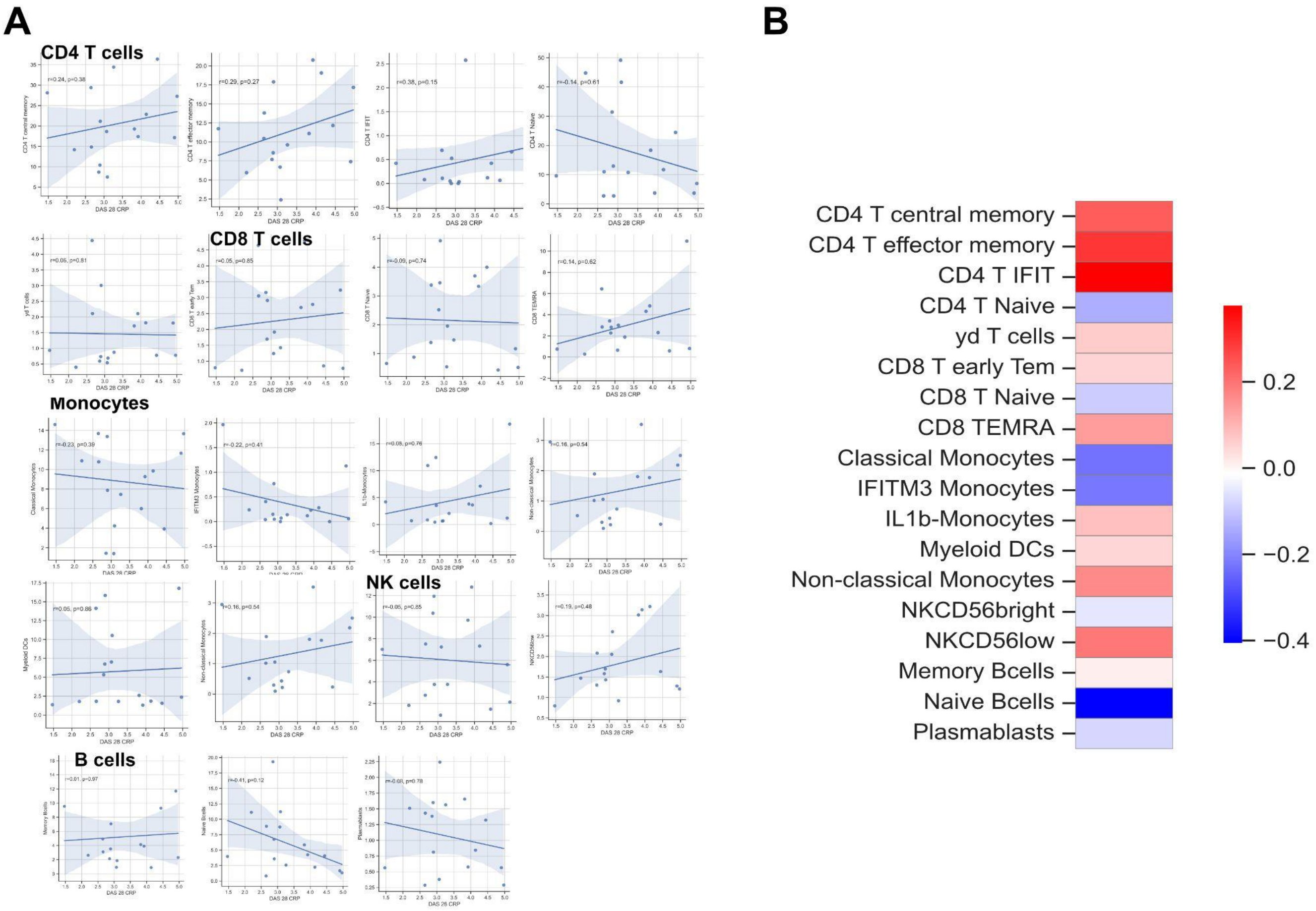

Figure S8 | Venn Diagram of the genes differentially expressed in RA low and high disease activity compared to controls. (FDR ≤ 0.05, log2(FC) ≥ log2(1.6), 0.08 ≤ mean expression< 4). RA, Rheumatoid Arthritis

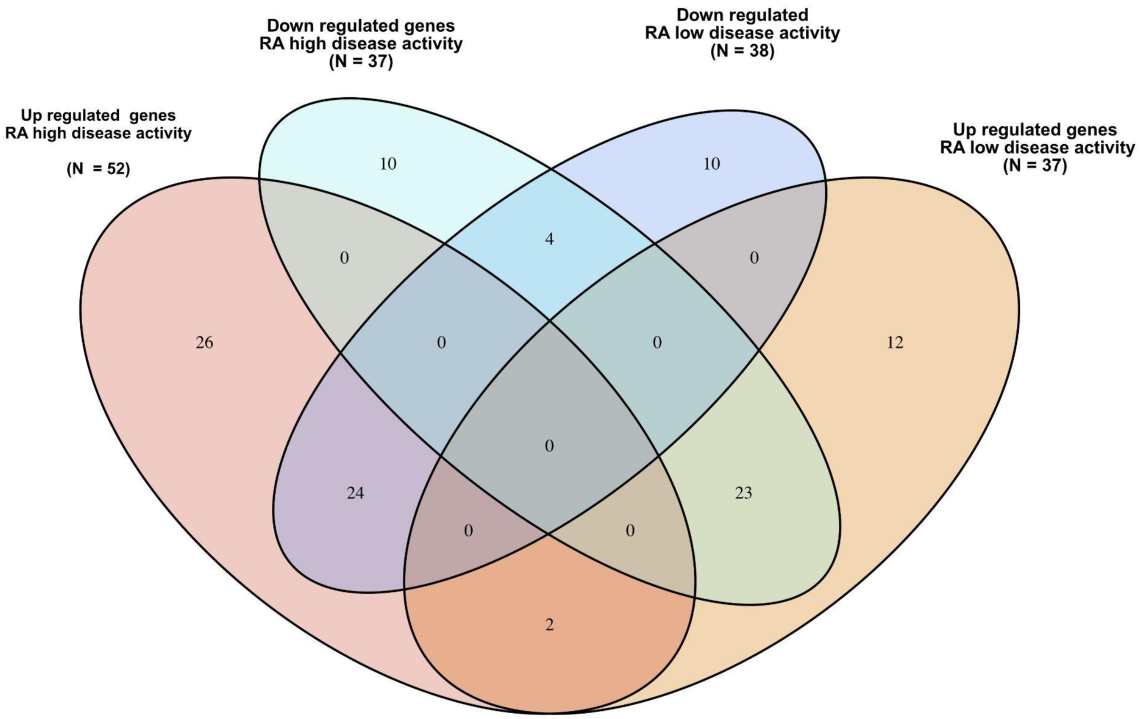

Figure S9 | Cell-cell communications between patients with low and high disease activity and matched controls. A. Heatmap representing the relative number of interactions between RA patients with high disease activity compared to controls B. Heatmap representing the relative number of interactions between RA patients with low disease activity compared to controls. DA Disease activity

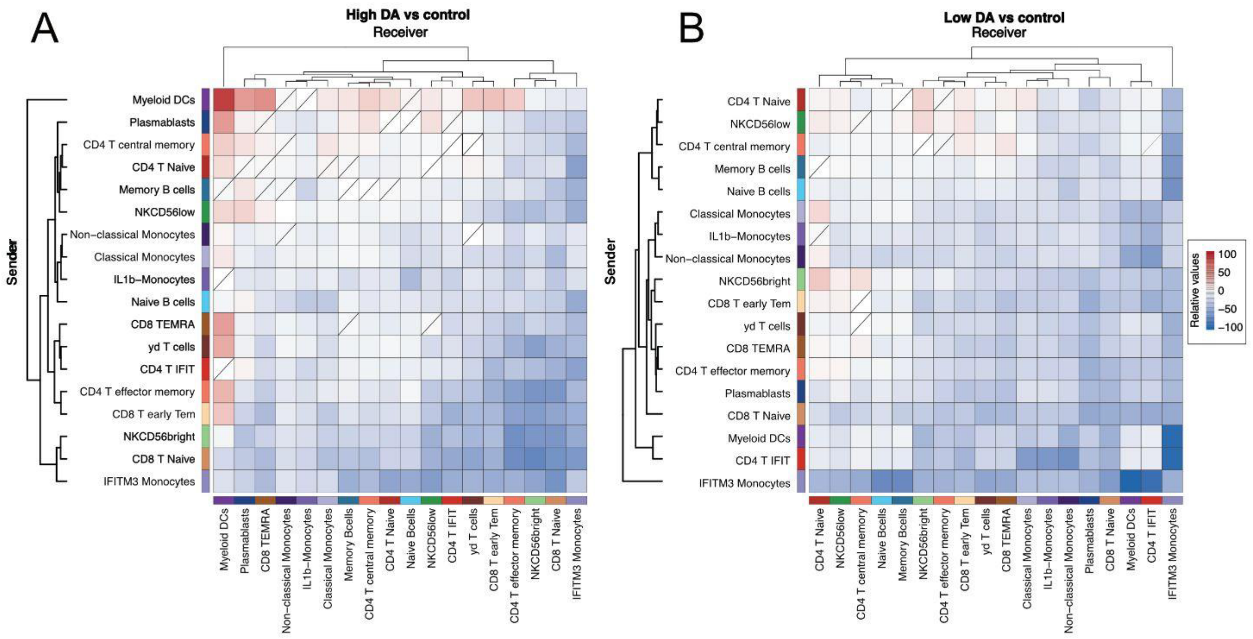

Figure S10 | A. Bar plots of all communication pathways based on interaction strength. Red are significantly more present in RA and blue are significantly more present in controls. B. Bar plots of all communication pathways based on number. DA, Disease Activity; RA, Rheumatoid Arthritis

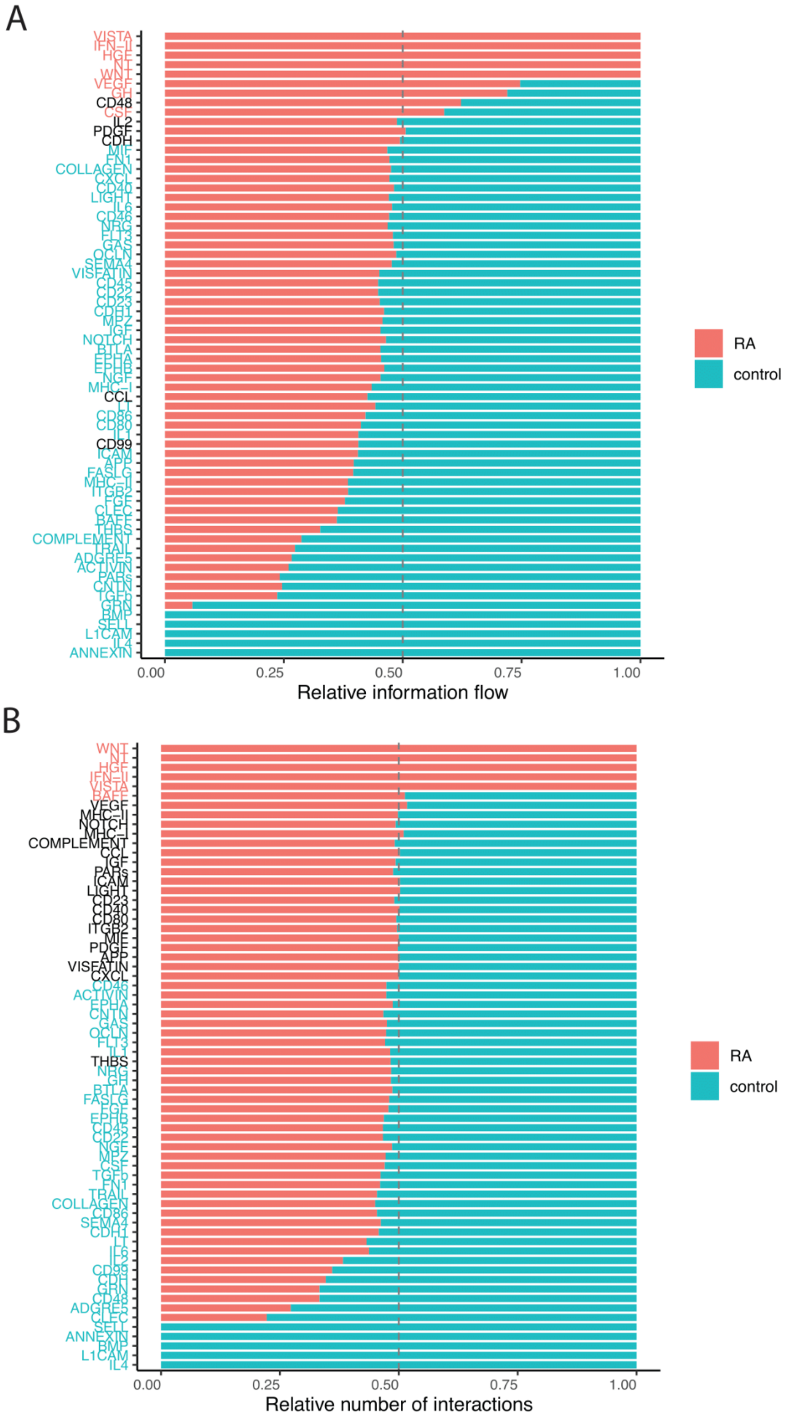

Figure S11 | A. Dot-plot of the relative contribution of communication pathways based on number of interactions between high and low disease activity compared to controls. B. Bar plots of all communication pathways based on number. Red are significantly more present in RA (low or high Disease activity) and blue are significantly more present in controls. C. Bar plots of all communication pathways based on interaction strength.DA, Disease Activity; RA, Rheumatoid Arthritis

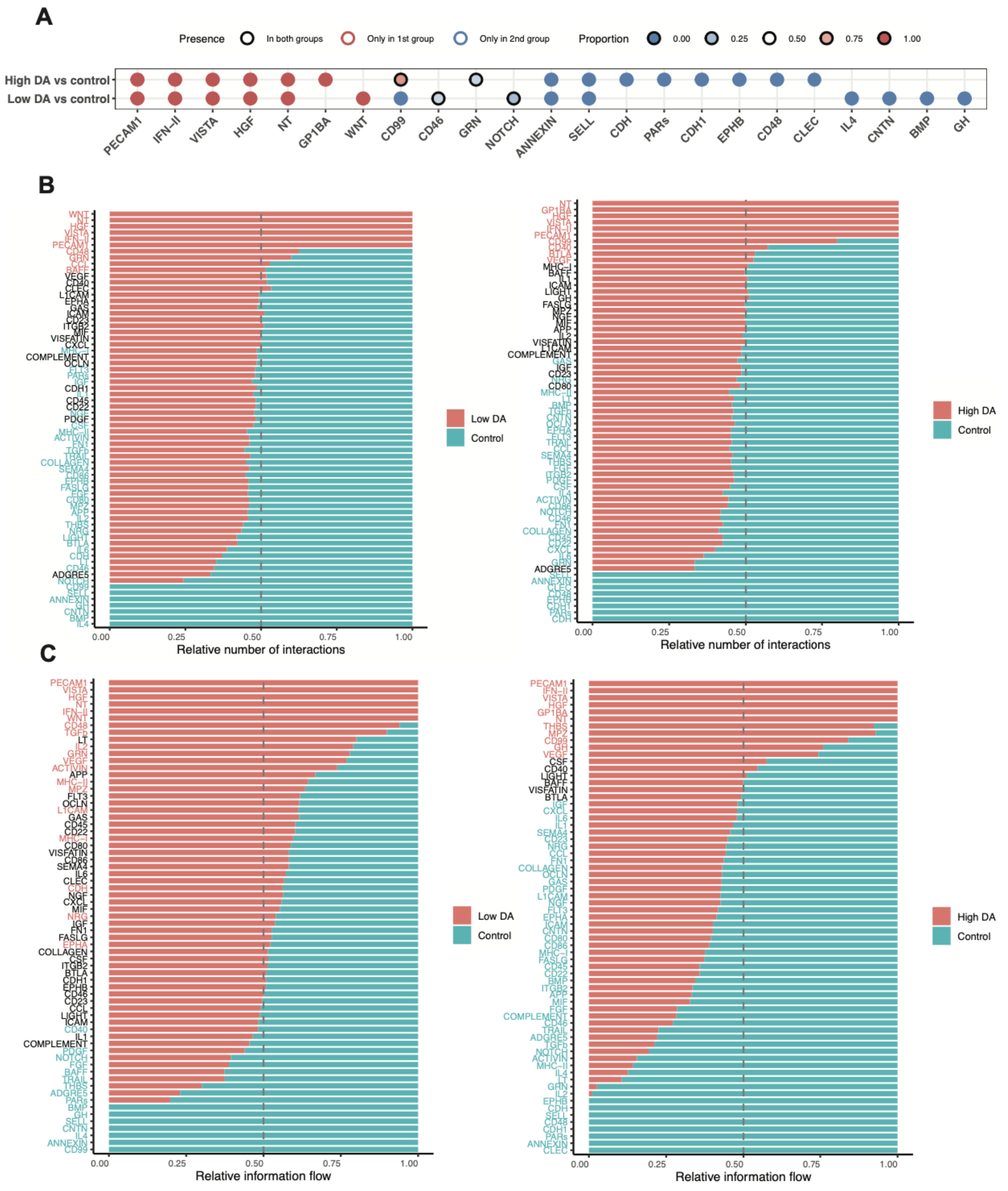

Figure S12 | A. Table of the ligand-receptor pairs that contribute to the communication for the PECAM1, HGF, IFN-II, NT and VISTA pathways. B. Bar plots showing the relative contribution of the ligand-receptor pairs that contribute to the communication for the VEGF and IL2 pathways for high and low disease activity and controls. DA, Disease activity; L, Ligand; R, Receptor.

A

Pathway	Ligand	Receptor
PECAM1	PECAM-1	PECAM1
HGF	HGF	MET
IFN-II	IFNG	IFNGR1+IFNGR2
NT	KLK3	NTRK1
VISTA	VSIR	IGSF11

B

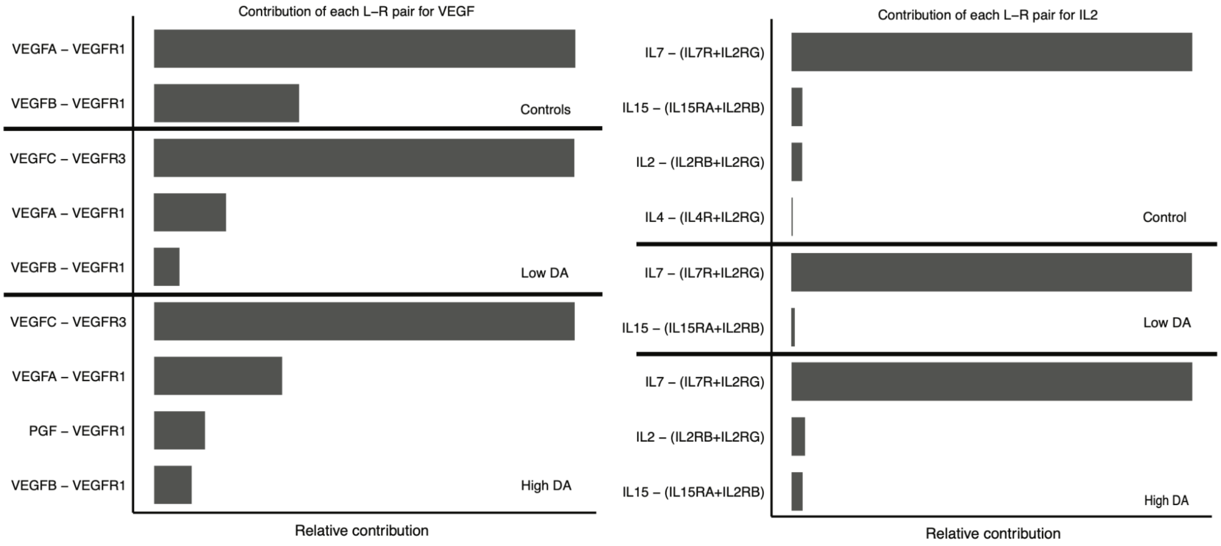

Figure S13 | Heatmaps of the relative importance of cells as senders and receivers for the VEGF, IL2, HGF, and PECAM1, NT signaling pathway network in high and low disease activity, and for VEGF and IL2 also controls. CD, Cluster differentiation; DC, :Dendritics cells, IFIT, Interferon Induced proteins with Tetratricopeptide repeats; IFITM, interferon-induced transmembrane; Tem, T effector memory; TEMRA, Terminally differentiated effector memory.

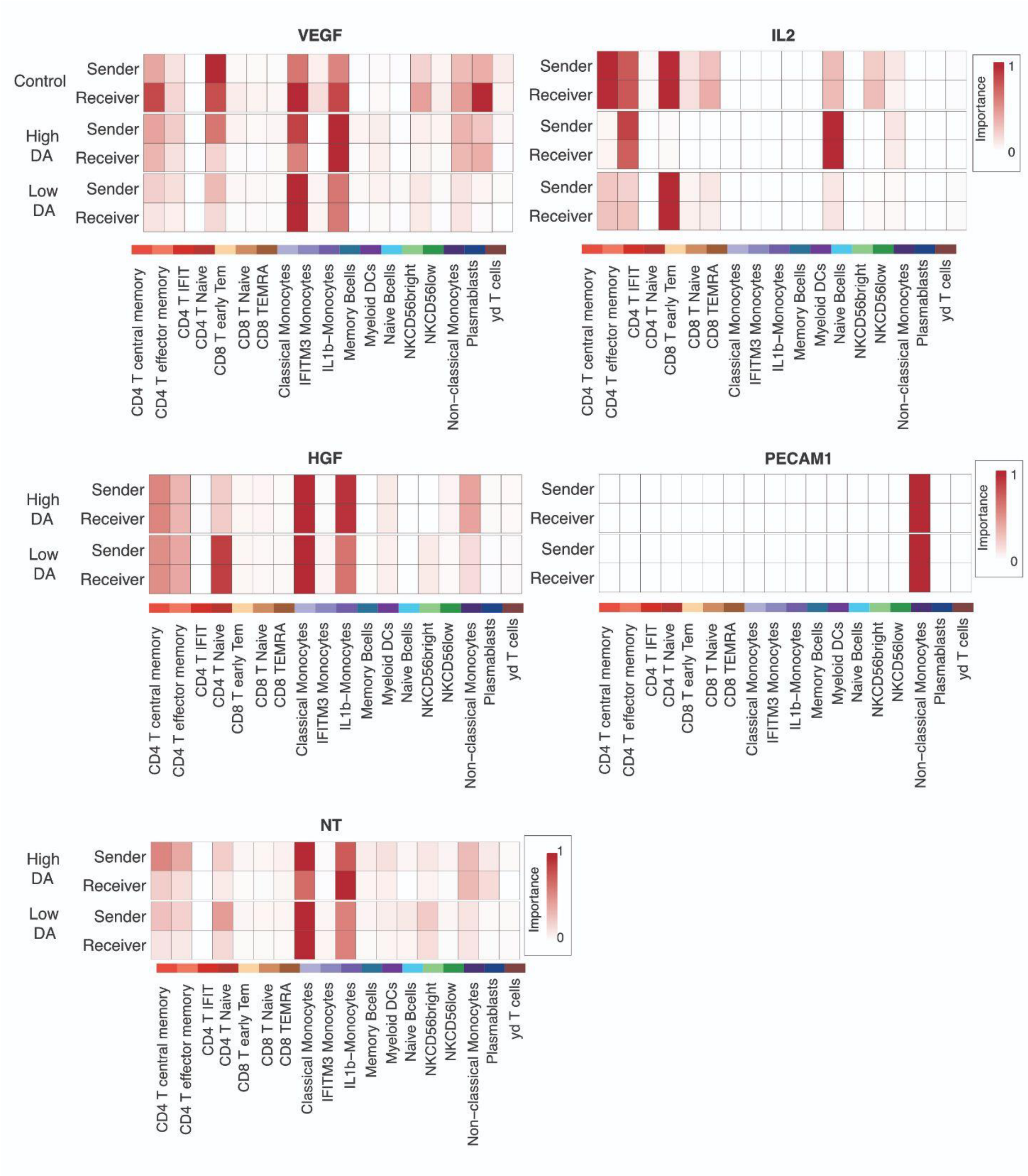

Figure S14 | Quality control metrics used to select filtering criteria. A. Histogram showing distribution of total number of sequencing reads, gene feature counts, and proportions of mitochondrial genes across sequenced cells. B. Violin plots showing the number of total reads and gene feature count identified in each cell, stratified by sequencing lane. C. Scatter plot showing the distribution of cells with high mitochondrial gene proportions, relative to total count and gene feature counts. D. Violin plots showing the proportion of mitochondrial, ribosomal, and hemoglobin associated genes identified in each cell, stratified by sequencing lane.

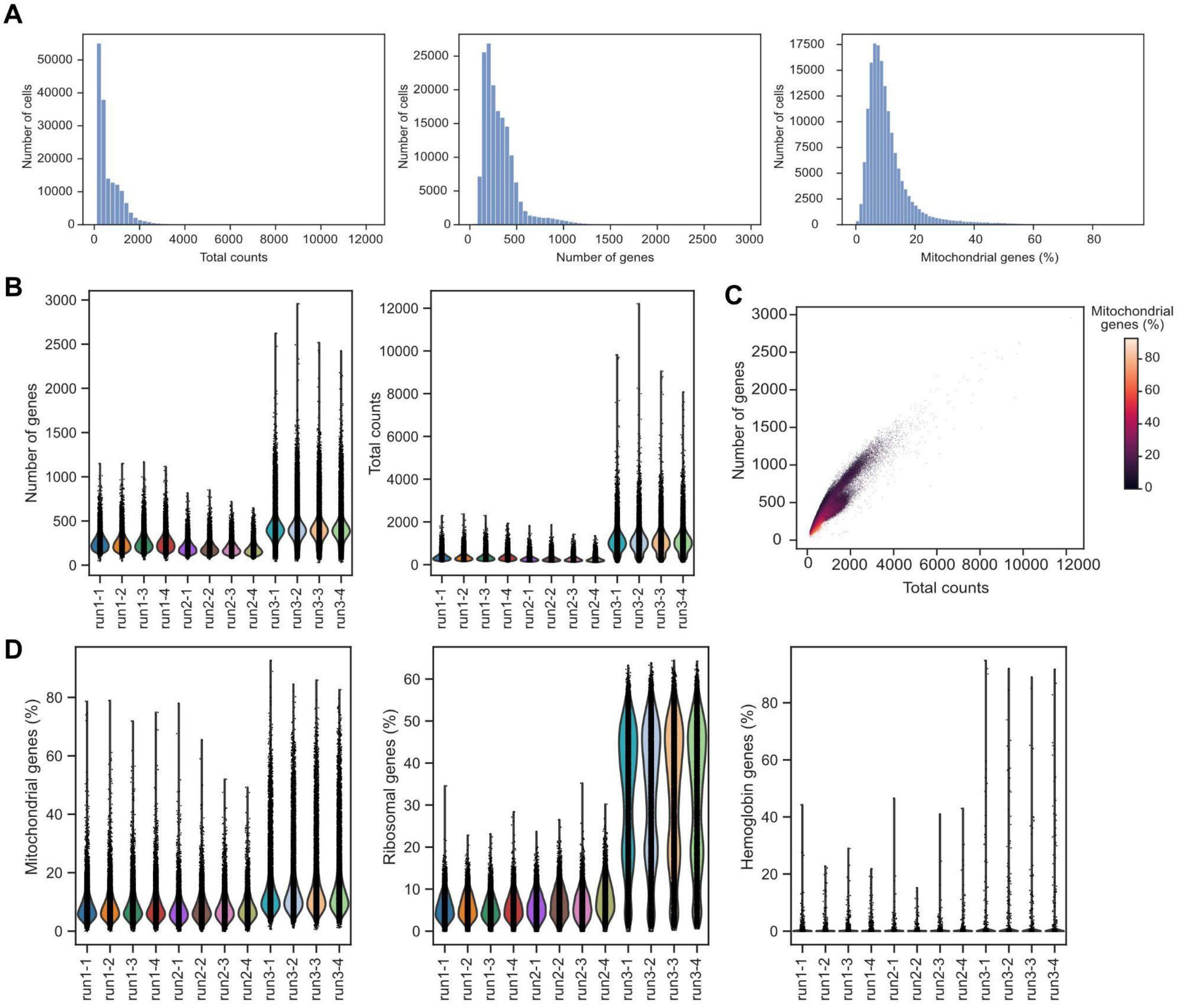

SUPPLEMENTARY TABLES:

Table S1 | Clinical characteristics of Rheumatoid arthritis patients and matched controls. p-value threshold for significance was set at ≤ 0.05. Student's t-test was conducted to analyze continuous variables. For categorical variables, a Chi-square test was performed. SD, standard deviation

Table S2 | List of differentially expressed genes between RA and matched controls and according to cell subtype. FDR ≤ 0.05, |log2(FC)| ≥ 1.6, 0.08 < base mean < 4. CD, Cluster differentiation; DCs, Dendritic cells; IFIT, Interferon Induced proteins with Tetratricopeptide repeats; IFITM, interferon-induced transmembrane; Tem, T effector memory; TEMRA, Terminally differentiated effector memory; RA, Rheumatoid Arthritis

Table S3 | Gene lists differentially expressed between RA and matched controls from pseudobulk analysis after adjustment on batch effect. FDR ≤ 0.05, |log2(FC)| ≥ 1.6, 0.08 < base mean < 4. CD, Cluster differentiation; DCs, Dendritic cells; FC, Fold change; FDR, False discovery rate; IFIT, Interferon Induced proteins with Tetratricopeptide repeats; IFITM, interferon-induced transmembrane; Tem, T effector memory; TEMRA, Terminally differentiated effector memory; RA, Rheumatoid Arthritis

Table S4 | Differentially regulated pathways between RA and matched controls. Gene ratio ≥ 0.15, qvalue ≤ 0.01, Count ≥ 5. CD, Cluster differentiation; DCs, Dendritic cells; FC, Fold change; FDR, False discovery rate; IFIT, Interferon Induced proteins with Tetratricopeptide repeats; IFITM, interferon-induced transmembrane; Tem, T effector memory; TEMRA, Terminally differentiated effector memory

Table S5 | Mean expression across controls, RA low- and high- disease activity across the 121 genes differentially expressed between RA and matched controls. FDR ≤ 0.05, |log2(FC)| ≥ 1.6, 0.08 < base mean < 4. FC, Fold change; FDR, False discovery rate; RA, Rheumatoid Arthritis

Table S6 | Quality control metrics of raw data stratified by lane. SD, standard deviation.

Table S7 | Quality control metrics of raw data, stratified by sample. SD, standard deviation.

Table S8 | Cell counts per sample, before and after preprocessing and filtering. SD, standard deviation.

Table S9 | Cell counts per lane, before and after preprocessing and filtering. SD, standard deviation.

Table S10 | Number of cells within each cell subset identified. CD: Cluster Differentiation; DCs: Dendritic Cells; IFIT: Interferon Induced proteins with Tetratricopeptide repeats; IFITM: Interferon-induced Transmembrane proteins; IL: Interleukin; IFN: Interferon; NK: Natural Killer; Tem: T Effector Memory; TEMRA: Terminally Differentiated Effector Memory.

Table S1 | Clinical characteristics between Rheumatoid arthritis patients and matched controls. The p-value threshold for significance was set at <0.05. Student’s t-test was conducted to analyze continuous variables. For categorical variables, a Chi-square test was performed. SD, standard deviation

		Controls (N = 18)	Rheumatoid arthritis (N = 18)	p-value	missing values (%)
**Sex** (%)	Female	12 (66.7 %)	12 (66.7 %)	ns	0
	Male	6 (33.33)	6 (33.33)		
**Age (years)** (mean (SD))		55.8 (16.8)	51.7 (15.3)	ns	11.1
**Race** (%)	Asian	2 (11.1 %)	2 (11.1 %)	ns	0
	Caucasian	15 (83.3 %)	15 (83.3 %)		
	Other	1 (5.6 %)	1 (5.6 %)		
**Ethnicity** (%)	Hispanic	1 (5.6 %)	1 (5.6 %)	ns	0
	Not Hispanic	17 (94.4 %)	17 (94.4 %)		

Table S2 | List of differentially expressed genes between RA and matched controls and according to cell subtype. FDR ≤ 0.05, |log2(FC)| ≥ 1.6, 0.08 < base mean < 4. CD, Cluster differentiation; DCs, Dendritics cells; IFIT, Interferon Induced proteins with Tetratricopeptide repeats; IFITM, interferon-induced transmembrane; Tem: T effector memory, TEMRA: Terminally differentiated effector memory. RA Rheumatoid Arthritis

Cell type	Cell subtype	Upregulated genes	Downregulated genes
**B cells**	Memory B cells	1	1
	Naive B cells	0	2
	Plasmablasts	1	1
**Monocytes**	Classical Monocytes	11	1
	IFITM3 Monocytes	11	1
	IL-1β Monocytes	21	3
	Myeloids DCs	2	4
	Non-classical Monocytes	15	16
**CD4 T cells**	CD4 IFIT	2	1
	CD4 Naive T cells	0	1
	CD4 T central memory	1	2
	CD4 T effector memory	1	0
	γδ T cells	7	11
**CD8 T cells**	CD8 T Naive	2	0
	CD8 T early Tem	8	14
	CD8 TEMRA	5	10
**NK cells**	NKCD56bright	1	2
	NKCD56low	0	0

Table S3 | Gene lists differentially expressed between RA and matched controls from pseudobulk analysis after adjustment on batch effect. FDR ≤ 0.05, |log2(FC)| ≥ 1.6, 0.08 < base mean < 4 CD, Cluster differentiation; DCs, Dendritic cells; FC, Fold change; FDR, False discovery rate ; IFIT, Interferon Induced proteins with Tetratricopeptide repeats; IFITM, interferon-induced transmembrane; Tem, T effector memory; TEMRA, Terminally differentiated effector memory; RA, Rheumatoid Arthritis

Cell subtype	gene	base mean	log2(FC)	p value	FDR
Classical Monocytes	PTGS2	0.099	-0.708	1.65E-09	6.42E-08
Classical Monocytes	THBS1	0.201	0.887	4.29E-10	1.91E-08
Classical Monocytes	ARRDC3	0.179	0.699	7.89E-13	4.36E-11
Classical Monocytes	CXCR4	0.123	0.679	2.59E-14	1.75E-12
Classical Monocytes	LMNA	0.206	0.707	2.76E-15	2.19E-13
Classical Monocytes	EAF1-AS1	0.099	1.069	8.73E-16	7.58E-14
Classical Monocytes	EGR1	0.567	0.925	7.96E-17	7.64E-15
Classical Monocytes	HSPA5	0.253	0.761	1.15E-22	1.61E-20
Classical Monocytes	EAF1	0.319	0.77	1.05E-22	1.60E-20
Classical Monocytes	MAFB	0.211	0.982	2.46E-29	1.12E-26
Classical Monocytes	TLE3	0.776	0.761	4.57E-47	4.16E-44
Classical Monocytes	MT2A	0.185	1.444	1.57E-62	2.86E-59
IL1b-Monocytes	SPN	0.083	-0.696	2.78E-05	0.00056038
IL1b-Monocytes	RCSD1	0.086	-0.688	6.05E-06	0.00015412
IL1b-Monocytes	GABPB1-AS1	0.123	-0.697	3.98E-07	1.43E-05
IL1b-Monocytes	PELI1	0.092	0.711	2.76E-07	1.05E-05
IL1b-Monocytes	MTPAP	0.157	0.685	1.04E-07	4.26E-06
IL1b-Monocytes	LGALS2	0.123	0.703	1.54E-10	9.49E-09
IL1b-Monocytes	CLEC12A	0.104	0.794	1.41E-10	8.94E-09

IL1b-Monocytes	HLA-DRB5	0.139	0.708	1.04E-10	6.96E-09
IL1b-Monocytes	SLA	0.082	0.978	6.67E-11	4.93E-09
IL1b-Monocytes	RFLNB	0.187	0.825	4.84E-11	3.97E-09
IL1b-Monocytes	EAF1-AS1	0.185	0.845	7.75E-12	6.87E-10
IL1b-Monocytes	IFITM2	0.119	0.801	1.48E-12	1.37E-10
IL1b-Monocytes	LMNA	0.223	0.928	6.36E-13	6.41E-11
IL1b-Monocytes	AGFG1	0.092	0.945	3.31E-13	3.50E-11
IL1b-Monocytes	ZBTB43	0.202	0.809	2.18E-13	2.42E-11
IL1b-Monocytes	SMAP2	0.143	0.785	1.70E-14	2.14E-12
IL1b-Monocytes	MT2A	0.098	0.972	1.74E-14	2.14E-12
IL1b-Monocytes	HSPA5	0.253	0.829	1.03E-14	1.43E-12
IL1b-Monocytes	ARRDC3	0.259	0.945	2.34E-17	4.32E-15
IL1b-Monocytes	EAF1	0.566	0.806	2.23E-24	5.49E-22
IL1b-Monocytes	RIT1	0.455	0.918	1.64E-31	5.18E-29
IL1b-Monocytes	VMP1	1.143	0.781	1.46E-34	6.45E-32
IL1b-Monocytes	EGR1	1.395	1.502	2.76E-41	2.04E-38
IL1b-Monocytes	TLE3	1.098	0.951	5.83E-49	1.29E-45
Myeloid DCs	TRBC1	0.133	-0.742	1.57E-09	3.88E-08
Myeloid DCs	MTRNR2L8	0.104	-0.875	1.12E-10	3.13E-09
Myeloid DCs	IL32	0.18	-0.751	1.13E-12	5.18E-11
Myeloid DCs	RIPOR2	0.195	-0.75	8.29E-15	4.87E-13
Myeloid DCs	BIRC3	0.105	1.162	8.60E-20	1.33E-17
Myeloid DCs	TXNIP	1.789	0.809	6.75E-82	8.32E-79
CD4 T central memory	CYBA	0.101	-0.691	3.44E-23	2.61E-21

CD4 T central memory	TRBC1	0.119	-0.761	4.76E-27	4.38E-25
CD4 T central memory	CRYBG1	0.089	0.975	2.36E-38	4.34E-36
CD8 T early Tem	CYTOR	0.09	0.77	0.00080387	0.02310796
CD8 T early Tem	POMP	0.094	0.698	0.00075527	0.02247245
CD8 T early Tem	NDUFB2	0.101	-0.682	0.00055605	0.01714656
CD8 T early Tem	MCL1	0.103	-0.695	0.00032015	0.01180386
CD8 T early Tem	BTG2	0.091	-0.768	0.000307	0.01157043
CD8 T early Tem	RNF149	0.096	0.83	0.00026488	0.01044719
CD8 T early Tem	GADD45B	0.099	-0.761	0.00023472	0.00947841
CD8 T early Tem	PSME2	0.086	-0.816	0.00019178	0.00813127
CD8 T early Tem	GRB2	0.104	-0.772	0.00018707	0.00813127
CD8 T early Tem	G3BP2	0.104	-0.795	0.00013568	0.00730483
CD8 T early Tem	EBP	0.09	0.941	0.00013783	0.00730483
CD8 T early Tem	TTC39C	0.167	-0.73	2.64E-05	0.00223818
CD8 T early Tem	HERPUD1	0.193	0.763	6.92E-06	0.00070256
CD8 T early Tem	SATB1	0.084	-1.064	5.62E-06	0.00063522
CD8 T early Tem	FGFBP2	0.093	1.051	4.55E-06	0.00055836
CD8 T early Tem	GZMH	0.221	0.791	1.27E-06	0.00019643
CD8 T early Tem	DUSP1	0.301	-0.715	3.77E-08	7.11E-06
CD8 T early Tem	KLRD1	0.331	0.753	2.12E-08	5.04E-06
CD8 T early Tem	FOS	0.442	-0.73	1.79E-08	5.04E-06
CD8 T early Tem	DUSP2	0.273	-0.754	1.26E-08	4.28E-06

CD8 T early Tem	KLRB1	0.533	-1.043	1.53E-13	8.67E-11
CD8 T early Tem	LTB	0.383	-0.998	4.77E-14	4.04E-11
Non-classical Monocytes	TMBIM4	0.084	-0.777	0.00181503	0.039543
Non-classical Monocytes	TMX1	0.083	-0.904	0.00170326	0.03784273
Non-classical Monocytes	PSMA4	0.101	-0.706	0.00140195	0.03243271
Non-classical Monocytes	TNF	0.085	0.934	0.0013204	0.03118921
Non-classical Monocytes	ZBTB43	0.098	0.744	0.00126258	0.03073397
Non-classical Monocytes	SELPLG	0.083	-0.91	0.00102424	0.02703997
Non-classical Monocytes	DNAJB14	0.108	0.703	0.00094052	0.02628576
Non-classical Monocytes	ZC3H4	0.108	-0.843	0.00083372	0.02413152
Non-classical Monocytes	CHORDC1	0.088	0.895	0.00042129	0.01575622
Non-classical Monocytes	FLI1	0.184	-0.754	0.00017407	0.00765894
Non-classical Monocytes	MPEG1	0.087	-0.934	0.00016335	0.00748079
Non-classical Monocytes	DUSP7	0.183	-0.785	0.00015376	0.00734126
Non-classical Monocytes	P2RX4	0.14	0.807	0.00013378	0.00652636
Non-classical Monocytes	JUN	0.284	0.688	9.12E-05	0.00499353
Non-classical Monocytes	ETNK1	0.177	-0.786	8.56E-05	0.00480424
Non-classical Monocytes	HCK	0.202	-0.722	7.78E-05	0.00459278
Non-classical Monocytes	RNF213	0.221	-0.7	2.82E-05	0.0019761
Non-classical Monocytes	GLUL	0.115	0.909	7.67E-06	0.00066235
Non-classical Monocytes	TNFSF13B	0.112	-1.044	7.60E-06	0.00066235
Non-classical Monocytes	EAF1	0.182	0.866	5.74E-06	0.00056047

Non-classical Monocytes	CXCR4	0.118	0.961	3.37E-06	0.000360<br>45
Non-classical Monocytes	MNDA	0.161	-1.043	1.28E-07	1.91E-05
Non-classical Monocytes	ARRDC3	0.335	0.837	2.03E-08	3.26E-06
Non-classical Monocytes	FGL2	0.358	-0.76	1.14E-08	1.97E-06
Non-classical Monocytes	DUSP6	0.293	0.801	7.02E-09	1.31E-06
Non-classical Monocytes	SPN	0.298	-0.886	2.12E-09	5.29E-07
Non-classical Monocytes	RIT1	0.277	0.914	1.18E-09	3.30E-07
Non-classical Monocytes	MT2A	0.306	0.944	4.34E-10	1.43E-07
Non-classical Monocytes	AHR	0.125	1.349	1.78E-10	7.97E-08
Non-classical Monocytes	IGSF6	0.249	-1.169	2.17E-11	1.62E-08
Non-classical Monocytes	EGR1	0.237	2.149	1.18E-17	2.65E-14
NKCD56bright	CMC1	0.146	-0.708	4.53E-12	8.70E-10
NKCD56bright	AREG	0.147	1.115	1.84E-25	1.23E-22
NKCD56bright	MYOM2	0.086	-2.695	4.26E-53	5.73E-50
CD4 T Naive	MTRNR2L8	0.084	-1.758	1.12E-79	1.43E-76
CD4 T effector memory	EGR1	0.429	1.011	3.36E-25	6.83E-23
Naive B cells	EGR1	0.308	-0.934	2.04E-09	1.23E-07
Naive B cells	MTRNR2L8	0.086	-1.1	1.03E-13	1.18E-11
Memory B cells	WHAMM	0.207	0.681	2.98E-12	3.47E-10
Memory B cells	MTRNR2L8	0.104	-1.522	7.10E-24	4.67E-21
CD8 T Naive	KLRD1	0.183	0.82	1.49E-06	0.000189<br>69
CD8 T Naive	TXNIP	0.865	0.806	7.58E-23	6.74E-20
yd T cells	HNRNPH3	0.1	0.832	0.001390<br>95	0.045032<br>03

yd T cells	IFNG	0.089	-1.202	0.001376 21	0.045032 03
yd T cells	STAG2	0.081	0.932	0.001201 73	0.040597 61
yd T cells	BTG2	0.11	-0.875	0.001059 12	0.036574 84
yd T cells	CTSW	0.146	-0.72	0.000884 18	0.031227 78
yd T cells	ISG15	0.092	-1.2	0.000487 83	0.020494 97
yd T cells	FKBP11	0.08	1.129	0.000487 98	0.020494 97
yd T cells	HERPUD1	0.232	0.684	0.000368 62	0.018478 78
yd T cells	TNF	0.09	-1.51	8.64E-05	0.007462 12
yd T cells	ANXA1	0.237	-0.76	7.91E-05	0.007328 1
yd T cells	IFIT2	0.114	-2.116	2.48E-05	0.003340 23
yd T cells	SYTL3	0.109	1.229	9.02E-06	0.001557 01
yd T cells	GZMA	0.154	-1.151	6.32E-07	0.000140 4
yd T cells	MT2A	0.213	1.421	3.55E-08	9.20E-06
yd T cells	TXNIP	0.98	0.707	9.01E-09	2.80E-06
yd T cells	PMAIP1	0.31	-1.311	1.90E-09	7.37E-07
yd T cells	TYROBP	0.132	-1.585	1.35E-09	6.97E-07
yd T cells	S100A4	0.564	-0.929	2.01E-10	3.12E-07
Plasmablasts	HLA-DRB5	0.181	0.76	0.000226 23	0.024250 75
Plasmablasts	MTRNR2L8	0.147	-1.427	1.56E-06	0.000477 94
CD8 TEMRA	TCF7	0.086	-0.704	0.001155 08	0.027255 9
CD8 TEMRA	GLS	0.092	-0.69	0.000937 05	0.023339 42
CD8 TEMRA	PRDM2	0.088	0.779	0.000178 78	0.006679 47

CD8 TEMRA	CORO1A	0.129	-0.708	6.09E-06	0.000303 47
CD8 TEMRA	CMC1	0.164	-0.731	1.89E-06	0.000105 72
CD8 TEMRA	CEMIP2	0.166	0.74	1.23E-06	7.87E-05
CD8 TEMRA	SYTL3	0.108	0.901	5.31E-07	3.97E-05
CD8 TEMRA	HERPUD1	0.174	0.874	1.03E-07	8.62E-06
CD8 TEMRA	FOS	0.302	-0.682	6.35E-08	6.10E-06
CD8 TEMRA	TRAC	0.129	-0.971	7.98E-09	8.25E-07
CD8 TEMRA	FGFBP2	0.238	0.741	7.05E-09	7.90E-07
CD8 TEMRA	FAM107B	0.106	-1.007	6.36E-09	7.90E-07
CD8 TEMRA	TRBC1	0.15	-0.984	8.79E-10	1.48E-07
CD8 TEMRA	GZMK	0.201	-0.98	6.18E-10	1.38E-07
CD8 TEMRA	JUNB	0.332	-0.794	2.36E-11	1.44E-08
IFITM3 Monocytes	TMA7	0.198	0.924	0.003291 29	0.048307 57
IFITM3 Monocytes	LRRK2	0.243	-1.141	0.003240 32	0.048307 57
IFITM3 Monocytes	AP2S1	0.222	-1.172	0.002813	0.042663 91
IFITM3 Monocytes	CEBPB	0.351	0.822	0.002484 99	0.038988 63
IFITM3 Monocytes	G0S2	0.45	-1.047	0.002160 69	0.035111 23
IFITM3 Monocytes	CCNL1	0.5	0.688	0.002059 15	0.034069 64
IFITM3 Monocytes	GUK1	0.223	-1.463	0.000917 36	0.018147 68
IFITM3 Monocytes	JUN	0.778	0.866	0.000793 65	0.017195 72
IFITM3 Monocytes	PTGS2	0.271	-1.657	0.000672 62	0.015302 04
IFITM3 Monocytes	CCL3	0.512	0.966	0.000663 24	0.015302 04
IFITM3 Monocytes	MPEG1	0.313	-1.144	0.000557 73	0.013356 22

IFITM3 Monocytes	CBX6	0.407	1.118	0.00051399	0.01335622
IFITM3 Monocytes	HLA-DQB1	0.598	-0.942	0.00024248	0.00689557
IFITM3 Monocytes	TLE3	0.615	1.092	0.00011724	0.00367897
IFITM3 Monocytes	LGALS1	0.827	0.681	9.63E-05	0.00324462
IFITM3 Monocytes	RIT1	0.238	1.625	5.66E-05	0.00214681
IFITM3 Monocytes	PSME2	0.435	-1.328	1.99E-05	0.00095114
IFITM3 Monocytes	MS4A7	0.312	-1.686	1.54E-05	0.00077682
IFITM3 Monocytes	FGL2	1.111	-0.864	3.51E-06	0.00024558
IFITM3 Monocytes	TXNIP	1.258	0.836	2.35E-06	0.00017841
IFITM3 Monocytes	HSPA5	0.283	2.307	5.85E-10	1.11E-07
CD4 T IFIT	CAPZA1	0.475	-0.817	0.00026236	0.02980862
CD4 T IFIT	TSC22D3	0.332	0.936	0.00015365	0.02269366
CD4 T IFIT	TXNIP	0.813	1.413	3.65E-13	5.38E-10

Table S4 | Pathways differentially up and downregulated between RA and matched controls. Gene ratio ≥ 0.15, qvalue ≤ 0.01, Count ≥ 5, CD, Cluster differentiation; DCs, Dendritic cells; FC, Fold change; FDR, False discovery rate ; IFIT, Interferon Induced proteins with Tetratricopeptide repeats; IFITM, interferon-induced transmembrane; Tem, T effector memory; TEMRA, Terminally differentiated effector memory

Cell type	Cell subtype	Pathways	ID	regulation	GeneRatio	Count	q-value
**B cells**	Naive B cells	antigen receptor-mediated signaling pathway	GO:0050851	up	0.216	8	2.67E-05
		activation of immune response	GO:0002253	up	0.243	9	2.91E-05
		immune response-activating cell surface receptor signaling pathway	GO:0002429	up	0.216	8	3.47E-05
		immune response-activating signal transduction	GO:0002757	up	0.216	8	3.47E-05
		B cell receptor signaling pathway	GO:0050853	up	0.162	6	3.98E-05
		immune response-regulating cell surface receptor signaling pathway	GO:0002768	up	0.216	8	4.56E-05
		B cell activation	GO:0042113	up	0.216	8	4.77E-05
		negative regulation of immune system process	GO:0002683	up	0.216	8	0.00027178
		lymphocyte mediated immunity	GO:0002449	up	0.189	7	0.00059505

		adaptive immune response based on somatic recombination of immune receptors built from immunoglobulin superfamily domains	GO:0002460	up	0.189	7	0.00059505
	Memory B cells	negative regulation of immune system process	GO:0002683	up	0.194	6	0.00597876
		regulation of translation	GO:0006417	down	0.158	9	0.00439578
**Monocytes**	Classical Monocytes	negative regulation of immune system process	GO:0002683	up	0.169	21	3.77E-09
	Non-classical Monocytes	response to mechanical stimulus	GO:0009612	up	0.16	8	0.00011157
		cytokine-mediated signaling pathway	GO:0019221	up	0.2	10	0.00041673
		mononuclear cell differentiation	GO:1903131	up	0.18	9	0.0013214
		lymphocyte differentiation	GO:0030098	up	0.16	8	0.00317799
		leukocyte proliferation	GO:0070661	down	0.226	7	0.00068047
		regulation of T cell activation	GO:0050863	down	0.226	7	0.00068047
		leukocyte cell-cell adhesion	GO:0007159	down	0.226	7	0.00071899
		regulation of leukocyte proliferation	GO:0070663	down	0.194	6	0.00071899
		negative regulation of leukocyte activation	GO:0002695	down	0.161	5	0.00218879
		negative regulation of cell activation	GO:0050866	down	0.161	5	0.00279676

		regulation of leukocyte cell-cell adhesion	GO:1903037	down	0.194	6	0.00279676
		regulation of lymphocyte proliferation	GO:0050670	down	0.161	5	0.0032455
		regulation of mononuclear cell proliferation	GO:0032944	down	0.161	5	0.0032455
		negative regulation of immune system process	GO:0002683	down	0.194	6	0.00473243
		mononuclear cell differentiation	GO:1903131	down	0.194	6	0.00541669
		regulation of cell-cell adhesion	GO:0022407	down	0.194	6	0.00541669
		lymphocyte proliferation	GO:0046651	down	0.161	5	0.00541669
	Myeloid DCs	RNA splicing	GO:0008380	up	0.183	17	1.93E-07
		regulation of mRNA metabolic process	GO:1903311	up	0.151	14	2.79E-07
		RNA splicing, via transesterification reactions with bulged adenosine as nucleophile	GO:0000377	up	0.151	14	2.96E-07
		mRNA splicing, via spliceosome	GO:0000398	up	0.151	14	2.96E-07
		RNA splicing, via transesterification reactions	GO:0000375	up	0.151	14	2.96E-07
		alpha-beta T cell activation	GO:0046631	down	0.152	12	1.22E-08
		maintenance of location	GO:0051235	down	0.165	13	1.29E-06
		leukocyte cell-cell adhesion	GO:0007159	down	0.165	13	8.44E-06
**CD4 T cells**	CD4 T central memory	RNA splicing	GO:0008380	up	0.164	20	1.41E-08

	CD4 T Naive	lymphocyte differentiation	GO:0030098	up	0.313	5	0.00284689
		mononuclear cell differentiation	GO:1903131	up	0.313	5	0.00339759
		T cell differentiation	GO:0030217	down	0.167	8	0.00029142
		mononuclear cell differentiation	GO:1903131	down	0.188	9	0.00070521
		regulation of hemopoiesis	GO:1903706	down	0.167	8	0.00143078
		lymphocyte differentiation	GO:0030098	down	0.167	8	0.00143078
		positive regulation of cytokine production	GO:0001819	down	0.167	8	0.00257768
	CD4 T effector memory	regulation of translation	GO:0006417	up	0.157	13	7.73E-05
	yd T cells	positive regulation of myeloid cell differentiation	GO:0045639	down	0.227	5	4.39E-05
		positive regulation of small molecule metabolic process	GO:0062013	down	0.227	5	0.00014305
		positive regulation of cytokine production	GO:0001819	down	0.318	7	0.00014305
		positive regulation of leukocyte differentiation	GO:1902107	down	0.227	5	0.0001963
		positive regulation of hemopoiesis	GO:1903708	down	0.227	5	0.0001963
		regulation of myeloid cell differentiation	GO:0045637	down	0.227	5	0.00032925
		response to virus	GO:0009615	down	0.273	6	0.00034246
		regulation of hemopoiesis	GO:1903706	down	0.273	6	0.00034246
**CD8 Tcells**	CD8 T Naive	response to mechanical stimulus	GO:0009612	down	0.208	5	0.00098074

Table S5 | Mean expression across controls, RA low and high disease activity , from the 121 genes differentially expressed between RA and matched controls from pseudobulk analysis after adjustment on batch effect. FDR ≤ 0.05, |log2(FC)| ≥ 1.6, 0.08 < base mean < 4 FC, Fold change; FDR, False discovery rate ; RA, Rheumatoid Arthritis

gene	Log2(FC) control group	Log2(FC) RA low disease activity	Log(2FC) RA high disease	FDR ≤ 0.05
AGFG1	-1.02	0.04	0.98	both
AHR	-0.31	-0.81	1.12	both
ANXA1	-1.15	0.67	0.47	RA low disease activity
AP2S1	0.26	0.84	-1.1	RA low disease activity
AREG	-0.27	1.11	-0.84	both
ARRDC3	0.03	-1.02	0.98	RA high disease activity
BIRC3	-1.15	0.65	0.51	RA high disease activity
BTG2	-0.29	-0.82	1.11	both
CAPZA1	0.42	-1.14	0.72	RA high disease activity
CBX6	0.05	-1.02	0.98	RA low disease activity
CCL3	0.25	0.85	-1.1	both
CCNL1	-0.57	-0.58	1.15	RA high disease activity
CEBPB	-0.76	1.13	-0.38	non significant
CEMIP2	0.34	-1.13	0.79	RA low disease activity
CHORDC1	-0.24	-0.86	1.1	both
CLEC12A	-0.55	1.15	-0.61	both
CMC1	1.07	-0.17	-0.91	both
CORO1A	0.42	0.72	-1.14	RA high disease activity
CRYBG1	-0.62	-0.54	1.15	RA low disease activity
CTSW	-0.24	1.1	-0.86	RA low disease activity

CXCR4	0.02	-1.01	0.99	RA low disease activity
CYBA	0.29	0.82	-1.11	both
CYTOR	-0.12	-0.93	1.06	RA high disease activity
DNAJB14	-0.04	-0.98	1.02	RA high disease activity
DUSP1	0.93	0.13	-1.06	RA low disease activity
DUSP2	0.86	0.24	-1.1	both
DUSP6	-0.47	-0.68	1.15	RA high disease activity
DUSP7	0.35	-1.13	0.78	RA low disease activity
EAF1	-0.25	-0.85	1.1	RA high disease activity
EAF1-AS1	-0.13	-0.93	1.06	RA high disease activity
EBP	-0.06	-0.97	1.03	RA low disease activity
EGR1	-0.31	-0.81	1.12	both
ETNK1	0.13	-1.06	0.93	RA low disease activity
FAM107B	-0.15	-0.92	1.07	both
FGFBP2	0.91	0.15	-1.07	both
FGL2	0.93	0.13	-1.06	RA high disease activity
FKBP11	-0.42	1.14	-0.72	RA low disease activity
FLI1	0.48	-1.15	0.67	RA high disease activity
FOS	0.94	-1.05	0.12	RA high disease activity
G0S2	0.57	-1.15	0.59	both
G3BP2	-0.3	-0.82	1.12	RA high disease activity
GABPB1-AS1	1.12	-0.8	-0.32	RA high disease activity
GADD45B	-1.15	0.7	0.45	RA low disease activity
GLS	-0.14	-0.92	1.06	RA high disease activity

GLUL	-0.47	1.15	-0.68	RA low disease activity
GRB2	0.52	-1.15	0.63	both
GUK1	0.25	0.85	-1.1	non significant
GZMA	0.72	0.42	-1.14	both
GZMH	-1.12	0.79	0.34	both
GZMK	0.63	0.52	-1.15	RA high disease activity
HCK	0.88	-1.09	0.21	non significant
HERPUD1	-0.1	-0.94	1.05	both
HLA-DQB1	0.28	0.83	-1.11	both
HLA-DRB5	0.08	0.96	-1.04	both
HNRNPH3	-1.07	0.91	0.16	non significant
HSPA5	-0.6	-0.56	1.15	both
IFIT2	0.3	-1.11	0.82	both
IFITM2	0.16	0.91	-1.07	both
IFNG	-0.17	-0.91	1.07	RA low disease activity
IGSF6	0.98	-1.02	0.04	both
IL32	0.28	0.83	-1.11	both
ISG15	0.63	0.52	-1.15	both
JUN	-0.3	-0.82	1.11	both
JUNB	0.8	-1.12	0.33	RA high disease activity
KLRB1	0.22	0.87	-1.09	both
KLRD1	0.1	-1.05	0.95	both
LGALS1	0.33	0.79	-1.12	both
LGALS2	-0.2	1.08	-0.89	RA low disease activity

LMNA	-0.47	-0.68	1.15	RA high disease activity
LRRK2	0.43	0.71	-1.14	RA high disease activity
LTB	0.16	0.91	-1.07	both
MAFB	-0.61	-0.54	1.15	both
MCL1	-0.71	-0.43	1.14	RA high disease activity
MNDA	0.17	0.9	-1.07	RA high disease activity
MPEG1	0.98	0.04	-1.02	both
MS4A7	1.14	-0.72	-0.42	both
MT2A	-0.26	1.1	-0.85	both
MTPAP	-0.15	-0.91	1.07	non significant
MTRNR2L8	0.89	0.2	-1.08	both
MYOM2	1.12	-0.33	-0.79	both
NDUFB2	0.47	0.68	-1.15	RA high disease activity
P2RX4	-0.11	-0.94	1.05	both
PELI1	-0.41	-0.73	1.14	RA low disease activity
PMAIP1	0.4	-1.14	0.74	both
POMP	-0.04	1.02	-0.98	RA high disease activity
PRDM2	-0.24	-0.86	1.1	both
PSMA4	0.36	0.77	-1.13	non significant
PSME2	0.53	0.62	-1.15	both
PTGS2	0.71	-1.14	0.43	RA low disease activity
RCSD1	1.13	-0.77	-0.36	RA high disease activity
RFLNB	-0.08	-0.96	1.04	RA high disease activity
RIPOR2	0.69	-1.15	0.46	RA high disease activity

RIT1	-0.19	-0.89	1.08	both
RNF149	0.05	-1.02	0.97	both
RNF213	-0.12	-0.93	1.05	RA high disease activity
S100A4	0.28	0.83	-1.11	RA low disease activity
SATB1	0.05	-1.02	0.97	both
SELPLG	0.76	0.37	-1.13	RA high disease activity
SLA	-0.27	-0.84	1.11	RA high disease activity
SMAP2	-0.33	1.12	-0.79	RA low disease activity
SPN	0.19	-1.08	0.89	both
STAG2	-0.3	-0.82	1.11	both
SYTL3	-1.01	0.03	0.99	RA low disease activity
TCF7	-0.22	-0.87	1.09	non significant
THBS1	0.18	-1.08	0.9	non significant
TLE3	-0.36	-0.77	1.13	both
TMA7	0.01	1	-1	non significant
TMBIM4	0.61	0.55	-1.15	non significant
TMX1	0.78	-1.13	0.35	RA high disease activity
TNF	0.05	-1.03	0.97	both
TNFSF13B	1.1	-0.24	-0.86	both
TRAC	0.3	0.81	-1.12	both
TRBC1	0.45	0.69	-1.15	both
TSC22D3	0.23	0.87	-1.09	RA low disease activity
TTC39C	0.21	-1.09	0.88	RA high disease activity
TXNIP	-0.14	1.06	-0.92	both

TYROBP	0.42	0.72	-1.14	both
VMP1	-0.41	-0.73	1.14	RA high disease activity
WHAMM	-0.38	-0.75	1.13	RA high disease activity
ZBTB43	-0.26	-0.84	1.11	RA high disease activity
ZC3H4	0.38	-1.13	0.76	RA high disease activity

Table S6 | Quality control metrics of raw data stratified by lane. SD: Standard deviation.

**nCount RNA**						
**Lane**	**Count (n)**	**mean**	**SD**	**min**	**50%**	**max**
run1-1	11753	380.71	183.08	136	334	1741
run1-2	11973	367.79	179.29	152	323	1851
run1-3	12166	371.74	181.87	158	325	1922
run1-4	11638	372.27	176.53	140	330	1785
run2-1	7853	289.78	135.22	135	257	1823
run2-2	7720	286.57	138.35	126	250	1866
run2-3	8144	281.7	133.1	125	246	1422
run2-4	7540	266.37	126.77	118	231	1231
run3-1	11328	1101.03	347.73	120	1074	2000
run3-2	12227	1087.27	368.07	128	1072	2000
run3-3	11620	1094.32	362.22	149	1076	2000
run3-4	11736	1104.69	351.28	175	1082	2000
**Genes (count) / nFeatures RNA**						
**Lane**	**Count (n)**	**mean**	**SD**	**min**	**50%**	**max**
run1-1	11753	269.16	101.51	107	247	977
run1-2	11973	260.53	99.68	110	240	973
run1-3	12166	263.35	101.23	103	241	999
run1-4	11638	264.23	99.41	103	245	960
run2-1	7853	206.64	75.08	100	191	816
run2-2	7720	204.95	78.26	100	187	851
run2-3	8144	200.71	74.6	100	183	718
run2-4	7540	188.44	70.38	100	172	646
run3-1	11328	443.92	133.37	100	415	996
run3-2	12227	435.15	139	100	409	996
run3-3	11620	434.49	136.34	103	408	982

run3-4	11736	440.15	136.24	116	410	989
**Mitochondrial genes (Count)**						
**Lane**	**Count (n)**	**mean**	**SD**	**min**	**50%**	**max**
run1-1	11753	29.05	18.29	0	25	206
run1-2	11973	28.18	17.59	1	24	205
run1-3	12166	27.8	17.16	0	24	194
run1-4	11638	28.23	17.67	1	24	195
run2-1	7853	19.72	12.52	0	17	132
run2-2	7720	20.42	12.89	0	18	134
run2-3	8144	19.95	12.44	0	17	118
run2-4	7540	19.69	11.96	0	17	167
run3-1	11328	117.52	51.64	2	107	388
run3-2	12227	117.62	54.43	2	107	385
run3-3	11620	118.15	55.18	1	107	377
run3-4	11736	119.67	54.63	6	108	372
**Mitochondrial genes (%)**						
**Lane**	**Count (n)**	**mean**	**SD**	**min**	**50%**	**max**
run1-1	11753	7.82	3.71	0	7.08	19.96
run1-2	11973	7.88	3.70	0.34	7.16	19.96
run1-3	12166	7.71	3.68	0	6.97	19.93
run1-4	11638	7.78	3.73	0.34	6.99	19.96
run2-1	7853	6.98	3.46	0	6.39	19.85
run2-2	7720	7.32	3.57	0	6.7	19.93
run2-3	8144	7.3	3.59	0	6.73	19.92
run2-4	7540	7.63	3.57	0	7.07	19.92
run3-1	11328	10.87	3.67	1.67	10.37	19.98
run3-2	12227	11	3.75	1.16	10.51	19.99

run3-3	11620	10.96	3.81	0.61	10.5	19.99
run3-4	11736	11.01	3.78	1.3	10.54	19.98
**Ribosomal genes (count)**						
**Lane**	**Count (n)**	**mean**	**SD**	**min**	**50%**	**max**
run1-1	11753	23.68	11.88	5	21	118
run1-2	11973	23.25	11.63	5	21	148
run1-3	12166	22	11.31	5	20	134
run1-4	11638	23.27	11.79	5	21	152
run2-1	7853	19.01	9.27	5	17	104
run2-2	7720	20.68	10.56	4	19	101
run2-3	8144	19.46	9.68	4	18	94
run2-4	7540	21.64	10.71	4	20	96
run3-1	11328	414.49	196.51	8	397	1120
run3-2	12227	412.67	205.37	7	395	1212
run3-3	11620	419.29	205.65	6	402	1143
run3-4	11736	419.5	200.04	8	405	1088
**Ribosomal genes (%)**						
**Lane**	**Count (n)**	**mean**	**SD**	**min**	**50%**	**max**
run1-1	11753	6.51	2.54	3	6.05	34.56
run1-2	11973	6.62	2.56	3	6.17	22.83
run1-3	12166	6.17	2.36	3	5.7	23.12
run1-4	11638	6.51	2.5	3	6.09	27.68
run2-1	7853	6.86	2.69	3	6.43	23.68
run2-2	7720	7.58	3.17	3	7.09	26.52
run2-3	8144	7.25	2.98	3	6.79	35.21
run2-4	7540	8.6	3.66	3.01	8.1	30.22
run3-1	11328	37.15	12.39	3.11	40.23	63.24

run3-2	12227	37.39	12.72	3.04	40.54	63.81
run3-3	11620	37.69	12.76	3.08	40.87	64.41
run3-4	11736	37.51	12.83	3.03	40.75	64.2
**Hemoglobin genes (Count)**						
**Lane**	**Count (n)**	**mean**	**SD**	**min**	**50%**	**max**
run1-1	11753	0.17	1.87	0	0	106
run1-2	11973	0.17	1.89	0	0	83
run1-3	12166	0.16	1.5	0	0	61
run1-4	11638	0.16	1.56	0	0	66
run2-1	7853	0.11	1.06	0	0	40
run2-2	7720	0.1	1.06	0	0	42
run2-3	8144	0.09	0.73	0	0	26
run2-4	7540	0.13	1.57	0	0	54
run3-1	11328	0.49	11.19	0	0	885
run3-2	12227	0.71	15.42	0	0	972
run3-3	11620	0.6	12.47	0	0	705
run3-4	11736	0.5	11.32	0	0	623
**Hemoglobin genes (%)**						
**Lane**	**Count (n)**	**mean**	**SD**	**min**	**50%**	**max**
run1-1	11753	0.044	0.448	0	0	27.53
run1-2	11973	0.047	0.544	0	0	22.75
run1-3	12166	0.045	0.43	0	0	18.89
run1-4	11638	0.047	0.495	0	0	19.19
run2-1	7853	0.039	0.38	0	0	17.65
run2-2	7720	0.037	0.395	0	0	15.16
run2-3	8144	0.035	0.286	0	0	8.26
run2-4	7540	0.047	0.612	0	0	24.77

run3-1	11328	0.041	0.794	0	0	48.9
run3-2	12227	0.063	1.296	0	0	74.03
run3-3	11620	0.047	0.874	0	0	38.92
run3-4	11736	0.041	0.8	0	0	43.84

Table S7 | Quality control metrics of raw data, stratified by sample. SD: Standard deviation.

**nCount RNA**							
**Sample**	**Batch**	**Count (n)**	**mean**	**SD**	**min**	**50%**	**max**
Control01	1	2629	318.94	141.37	152	285	1460
Control03	1	2658	315.16	128.49	136	287	1991
Control04	1	4906	342.56	146.63	157	309	1354
Control05	1	5869	394.00	195.94	152	346	2298
Control06	1	2575	350.34	151.03	156	313	1635
Control07	1	4143	369.98	174.64	154	329	1716
Control07	2	3004	256.94	110.05	119	230	1178
Control07	3	6247	1278.73	646.78	136	1152	7971
Control08	1	5172	370.47	171.95	156	329	2292
Control09	2	6381	308.01	149.68	118	270	1453
Control10	2	6293	280.92	135.09	114	245	1296
Control15	2	5003	289.86	147.37	117	247	1823
Control16	2	5413	261.13	116.79	116	234	1866
Control17	3	2610	963.35	537.13	105	935.5	4681
Control18	3	2669	877.87	580.99	116	815	6448
Control19	3	4001	1014.73	557.81	139	953	5855
Control20	3	4173	1309.46	742.19	142	1129	6496
Control21	3	5235	1212.00	630.01	131	1109	9049
Control22	3	4091	1116.93	560.84	131	1009	5666
Control23	3	5337	1146.28	580.06	138	1054	8076
RA01	1	5368	398.32	209.91	156	341	2082
RA03	1	3498	364.33	177.91	153	318	1721
RA04	1	3584	334.25	141.68	158	302	1563
RA05	1	4937	433.76	224.95	155	372	1929
RA06	1	3983	360.03	167.01	151	320	1987
RA07	1	3131	381.87	184.61	124	335	2369

RA08	1	6878	389.06	187.76	140	344	1649
RA09	2	3520	294.89	139.00	119	261	1231
RA10	2	2999	252.90	114.30	117	224	1422
RA15	2	4783	273.72	128.05	115	242	1275
RA16	3	3135	1005.22	561.68	128	1014	7437
RA17	3	3531	983.82	559.99	120	913	5601
RA18	3	2912	1197.38	564.96	144	1124	5460
RA19	3	3699	1172.18	656.26	113	1016	9706
RA20	3	3427	1276.46	586.04	115	1196	6501
RA21	3	3213	926.54	520.41	114	932	8000
RA22	3	4448	997.27	591.97	105	951	9819
RA23	3	5051	1254.59	737.29	138	1107	12207
**Genes (count) / nFeatures RNA**							
**Lane**	**Batch**	**Count (n)**	**mean**	**SD**	**min**	**50%**	**max**
Control01	1	2629	230.94	80.20	72	215	741
Control03	1	2658	230.08	77.66	54	216	1012
Control04	1	4906	246.40	86.25	56	229	772
Control05	1	5869	272.40	107.59	91	250	1167
Control06	1	2575	250.42	91.01	63	232	803
Control07	1	4143	261.90	100.51	90	241	952
Control07	2	3004	186.47	65.77	74	172	674
Control07	3	6247	510.08	240.41	73	437	2178
Control08	1	5172	268.26	101.89	98	247	1149
Control09	2	6381	214.38	84.11	78	196	780
Control10	2	6293	199.32	77.71	69	181	725
Control15	2	5003	197.37	76.06	67	180	851
Control16	2	5413	188.63	68.36	80	174	759
Control17	3	2610	381.90	188.48	50	367	1564

Control18	3	2669	359.09	202.93	31	352	1597
Control19	3	4001	397.90	189.20	44	366	1612
Control20	3	4173	572.57	293.05	75	471	2016
Control21	3	5235	484.94	226.43	71	425	2407
Control22	3	4091	431.60	189.53	40	390	1669
Control23	3	5337	479.55	209.58	34	437	2141
RA01	1	5368	270.89	108.97	86	247	1150
RA03	1	3498	254.56	98.07	48	236	977
RA04	1	3584	244.62	85.42	79	228	814
RA05	1	4937	291.16	119.32	73	267	1116
RA06	1	3983	253.38	94.42	63	234	1147
RA07	1	3131	266.87	102.55	47	246	1136
RA08	1	6878	271.99	105.18	103	252	1023
RA09	2	3520	206.82	78.39	67	190	646
RA10	2	2999	184.48	66.50	77	169	678
RA15	2	4783	194.31	72.57	78	179	769
RA16	3	3135	372.69	175.46	49	379	2125
RA17	3	3531	393.53	193.66	64	382	1724
RA18	3	2912	453.10	197.27	73	408	1540
RA19	3	3699	472.60	226.12	73	405	2476
RA20	3	3427	476.77	207.61	45	417	1825
RA21	3	3213	335.63	143.74	64	341	2332
RA22	3	4448	400.63	203.51	49	381	2622
RA23	3	5051	513.56	264.02	68	434	2957
**Mitochondrial genes (Count)**							
**Lane**	**Batch**	**Count (n)**	**mean**	**SD**	**min**	**50%**	**max**
Control01	1	2629	29.10	20.94	0	24	226
Control03	1	2658	27.33	19.20	1	22	194

Control04	1	4906	26.75	19.04	1	22	299
Control05	1	5869	32.37	21.73	0	27	335
Control06	1	2575	35.41	22.31	2	30	194
Control07	1	4143	29.77	20.84	1	24	245
Control07	2	3004	20.91	14.91	0	17	150
Control07	3	6247	151.79	88.42	3	129	1640
Control08	1	5172	28.99	20.49	1	24	242
Control09	2	6381	20.98	14.66	0	17	162
Control10	2	6293	23.26	16.01	0	19	221
Control15	2	5003	23.20	15.38	0	20	274
Control16	2	5413	20.05	13.39	0	17	194
Control17	3	2610	136.37	80.59	6	113	1083
Control18	3	2669	138.24	104.37	5	114	3290
Control19	3	4001	140.34	79.12	6	120	1203
Control20	3	4173	135.36	77.68	1	116	777
Control21	3	5235	145.53	87.06	3	123	1987
Control22	3	4091	147.45	76.61	7	129	762
Control23	3	5337	134.87	78.30	4	114	782
RA01	1	5368	32.55	21.40	1	27	230
RA03	1	3498	33.38	22.40	1	28	209
RA04	1	3584	26.31	19.90	0	21	242
RA05	1	4937	30.32	21.53	1	25	238
RA06	1	3983	28.66	20.34	1	23	227
RA07	1	3131	30.72	21.60	1	25	225
RA08	1	6878	34.80	23.95	1	29	697
RA09	2	3520	22.69	16.04	0	19	230
RA10	2	2999	15.68	12.19	0	13	154
RA15	2	4783	22.84	14.34	0	20	167
RA16	3	3135	150.12	81.86	6	131	1553

RA17	3	3531	158.46	84.63	2	139	1653
RA18	3	2912	133.02	72.04	9	113	528
RA19	3	3699	117.10	82.35	8	91	1133
RA20	3	3427	137.21	78.35	7	117	1134
RA21	3	3213	119.19	73.45	4	98	1057
RA22	3	4448	134.27	79.03	7	111	1086
RA23	3	5051	176.41	96.85	8	154	1585
**Mitochondrial genes (%)**							
**Lane**	**Batch**	**Count (n)**	**mean**	**SD**	**min**	**50%**	**max**
Control01	1	2629	9.38	5.88	0.00	7.89	57.14
Control03	1	2658	8.88	5.68	0.49	7.34	69.09
Control04	1	4906	8.02	5.00	0.32	6.79	74.88
Control05	1	5869	8.56	4.74	0.00	7.48	53.59
Control06	1	2575	10.48	6.05	1.04	9.02	74.69
Control07	1	4143	8.26	4.80	0.42	7.07	55.34
Control07	2	3004	8.29	4.98	0.00	7.23	49.82
Control07	3	6247	12.89	7.46	0.68	10.94	82.64
Control08	1	5172	8.00	4.66	0.56	6.86	52.06
Control09	2	6381	7.13	4.40	0.00	6.12	50.25
Control10	2	6293	8.57	4.87	0.00	7.50	67.58
Control15	2	5003	8.42	4.64	0.00	7.43	65.48
Control16	2	5413	7.92	4.53	0.00	7.04	49.24
Control17	3	2610	17.65	12.72	1.02	12.90	73.18
Control18	3	2669	21.08	15.04	2.13	14.91	88.92
Control19	3	4001	16.75	11.63	2.04	12.48	72.16
Control20	3	4173	11.99	7.93	0.61	9.58	77.61
Control21	3	5235	13.17	7.71	1.10	11.06	69.05
Control22	3	4091	14.81	8.79	0.67	12.23	84.50

Control23	3	5337	12.94	7.84	1.52	10.77	76.29
RA01	1	5368	8.60	4.73	0.36	7.43	55.34
RA03	1	3498	9.54	5.70	0.49	8.15	78.92
RA04	1	3584	8.06	5.30	0.00	6.71	72.02
RA05	1	4937	7.30	4.36	0.26	6.19	59.02
RA06	1	3983	8.29	5.24	0.51	6.98	68.87
RA07	1	3131	8.30	4.90	0.38	7.18	78.65
RA08	1	6878	9.27	4.87	0.71	8.15	48.68
RA09	2	3520	7.97	4.72	0.00	6.96	77.97
RA10	2	2999	6.31	4.06	0.00	5.47	47.83
RA15	2	4783	8.69	4.67	0.00	7.76	47.67
RA16	3	3135	19.29	13.89	2.46	13.56	79.62
RA17	3	3531	19.95	13.09	1.67	14.93	78.60
RA18	3	2912	12.55	8.27	1.30	10.22	69.04
RA19	3	3699	10.93	7.34	1.38	8.89	80.03
RA20	3	3427	11.84	7.52	2.49	9.73	92.65
RA21	3	3213	16.99	13.28	1.46	11.79	79.03
RA22	3	4448	17.02	12.58	1.68	12.26	85.92
RA23	3	5051	15.98	8.99	2.39	13.32	76.56
**Ribosomal genes (count)**							
**Lane**	**Batch**	**Count (n)**	**mean**	**SD**	**min**	**50%**	**max**
Control01	1	2629	17.86	11.17	0	16	125
Control03	1	2658	21.29	11.44	0	20	115
Control04	1	4906	19.88	11.90	0	18	100
Control05	1	5869	19.81	11.64	0	18	103
Control06	1	2575	17.92	10.81	0	16	84
Control07	1	4143	20.59	12.24	0	19	133
Control07	2	3004	17.73	9.72	0	17	71

Control07	3	6247	416.90	241.92	2	394	1995
Control08	1	5172	18.46	12.23	0	16	99
Control09	2	6381	17.65	11.27	0	16	96
Control10	2	6293	16.90	10.79	0	15	80
Control15	2	5003	17.58	10.38	0	16	88
Control16	2	5413	19.21	10.53	0	18	104
Control17	3	2610	338.22	247.08	2	353	1437
Control18	3	2669	287.40	259.99	1	244	1689
Control19	3	4001	361.15	245.40	0	354	2669
Control20	3	4173	344.42	211.36	3	327	2061
Control21	3	5235	402.11	232.53	2	396	2643
Control22	3	4091	386.04	228.13	4	362	1679
Control23	3	5337	378.88	233.38	2	359	2261
RA01	1	5368	20.25	12.57	0	18	148
RA03	1	3498	23.33	12.14	0	22	101
RA04	1	3584	20.61	11.28	0	19	105
RA05	1	4937	23.83	14.02	0	22	152
RA06	1	3983	18.34	11.54	0	16	143
RA07	1	3131	20.67	12.56	0	19	109
RA08	1	6878	20.12	12.44	0	18	124
RA09	2	3520	18.77	11.48	0	17	101
RA10	2	2999	18.85	10.23	0	18	94
RA15	2	4783	17.43	10.67	0	15	84
RA16	3	3135	385.29	287.68	2	389	2062
RA17	3	3531	333.26	259.21	0	304	2307
RA18	3	2912	447.84	246.90	3	441	2360
RA19	3	3699	402.34	242.05	0	360	2669
RA20	3	3427	479.22	257.51	1	457	2317
RA21	3	3213	398.88	299.88	3	409	2449

RA22	3	4448	351.47	263.26	2	341	2982
RA23	3	5051	361.80	247.30	4	325	2979
**Ribosomal genes (%)**							
**Lane**	**Batch**	**Count (n)**	**mean**	**SD**	**min**	**50%**	**max**
Control01	1	2629	5.72	2.94	0.00	5.41	25.26
Control03	1	2658	6.88	3.08	0.00	6.72	34.56
Control04	1	4906	5.88	2.77	0.00	5.51	22.83
Control05	1	5869	5.25	2.49	0.00	4.93	17.86
Control06	1	2575	5.18	2.58	0.00	4.97	17.34
Control07	1	4143	5.82	2.91	0.00	5.47	19.65
Control07	2	3004	7.24	3.70	0.00	7.00	23.77
Control07	3	6247	33.04	14.86	0.72	35.86	63.19
Control08	1	5172	5.03	2.62	0.00	4.64	18.66
Control09	2	6381	6.02	3.52	0.00	5.44	35.21
Control10	2	6293	6.25	3.48	0.00	5.74	24.29
Control15	2	5003	6.46	3.39	0.00	6.07	23.61
Control16	2	5413	7.61	3.58	0.00	7.35	26.80
Control17	3	2610	31.10	17.24	0.75	35.80	64.41
Control18	3	2669	26.58	18.08	0.23	26.99	61.34
Control19	3	4001	32.92	15.41	0.00	36.60	61.93
Control20	3	4173	27.08	12.79	0.95	26.51	59.23
Control21	3	5235	32.60	12.97	0.69	35.76	57.42
Control22	3	4091	33.67	13.32	1.07	36.31	60.33
Control23	3	5337	32.10	13.67	0.76	35.10	61.26
RA01	1	5368	5.32	2.62	0.00	4.83	27.13
RA03	1	3498	6.83	3.20	0.00	6.66	20.45
RA04	1	3584	6.29	2.75	0.00	6.15	18.09
RA05	1	4937	5.90	3.01	0.00	5.43	21.91

RA06	1	3983	5.23	2.65	0.00	4.73	22.41
RA07	1	3131	5.65	2.99	0.00	5.17	28.38
RA08	1	6878	5.31	2.63	0.00	4.93	23.12
RA09	2	3520	6.69	3.65	0.00	6.19	23.39
RA10	2	2999	7.76	3.61	0.00	7.41	26.49
RA15	2	4783	6.58	3.38	0.00	6.25	25.65
RA16	3	3135	32.64	17.84	0.74	39.49	61.84
RA17	3	3531	29.64	16.09	0.00	33.23	59.74
RA18	3	2912	36.75	14.54	1.40	40.90	63.72
RA19	3	3699	34.54	14.09	0.00	35.56	63.81
RA20	3	3427	37.13	14.06	0.31	39.86	62.25
RA21	3	3213	36.40	18.80	0.67	43.16	64.34
RA22	3	4448	31.49	16.56	0.58	35.62	60.13
RA23	3	5051	28.53	13.79	0.58	26.70	58.98
**Hemoglobin genes (count)**							
**Lane**	**Batch**	**Count (n)**	**mean**	**SD**	**min**	**50%**	**max**
Control01	1	2629	0.13	1.49	0	0	57
Control03	1	2658	0.19	2.03	0	0	55
Control04	1	4906	0.21	2.22	0	0	78
Control05	1	5869	0.15	1.56	0	0	72
Control06	1	2575	0.13	1.11	0	0	23
Control07	1	4143	0.19	1.72	0	0	59
Control07	2	3004	0.10	1.10	0	0	35
Control07	3	6247	0.78	22.26	0	0	1580
Control08	1	5172	0.19	2.06	0	0	66
Control09	2	6381	0.13	1.77	0	0	101
Control10	2	6293	0.13	1.54	0	0	75
Control15	2	5003	0.09	1.10	0	0	59

Control16	2	5413	0.11	1.31	0	0	54
Control17	3	2610	2.02	44.46	0	0	1718
Control18	3	2669	1.34	33.11	0	0	1300
Control19	3	4001	1.19	27.36	0	0	1110
Control20	3	4173	0.93	24.06	0	0	1428
Control21	3	5235	1.45	32.32	0	0	1514
Control22	3	4091	0.98	25.78	0	0	1409
Control23	3	5337	1.78	41.46	0	0	1509
RA01	1	5368	0.17	2.20	0	0	106
RA03	1	3498	0.17	2.62	0	0	102
RA04	1	3584	0.15	1.30	0	0	36
RA05	1	4937	0.27	1.94	0	0	77
RA06	1	3983	0.18	1.88	0	0	71
RA07	1	3131	0.18	1.30	0	0	39
RA08	1	6878	0.13	1.45	0	0	56
RA09	2	3520	0.14	1.33	0	0	48
RA10	2	2999	0.12	1.18	0	0	40
RA15	2	4783	0.14	3.20	0	0	207
RA16	3	3135	1.23	28.06	0	0	1426
RA17	3	3531	0.38	7.02	0	0	273
RA18	3	2912	0.46	8.62	0	0	406
RA19	3	3699	0.80	17.84	0	0	905
RA20	3	3427	1.13	21.46	0	0	954
RA21	3	3213	1.37	38.09	0	0	1512
RA22	3	4448	2.19	49.58	0	0	1624
RA23	3	5051	1.81	40.45	0	0	1792
**Hemoglobin genes (%)**							
**Lane**	**Batch**	**Count (n)**	**mean**	**SD**	**min**	**50%**	**max**

Control01	1	2629	0.04	0.47	0.00	0.00	18.87
Control03	1	2658	0.07	0.82	0.00	0.00	23.08
Control04	1	4906	0.06	0.59	0.00	0.00	18.89
Control05	1	5869	0.04	0.52	0.00	0.00	26.57
Control06	1	2575	0.04	0.32	0.00	0.00	5.71
Control07	1	4143	0.06	0.54	0.00	0.00	20.27
Control07	2	3004	0.04	0.57	0.00	0.00	19.31
Control07	3	6247	0.06	1.40	0.00	0.00	90.08
Control08	1	5172	0.06	0.66	0.00	0.00	22.75
Control09	2	6381	0.05	0.70	0.00	0.00	42.98
Control10	2	6293	0.05	0.58	0.00	0.00	24.51
Control15	2	5003	0.04	0.64	0.00	0.00	40.97
Control16	2	5413	0.05	0.60	0.00	0.00	24.77
Control17	3	2610	0.17	3.16	0.00	0.00	91.97
Control18	3	2669	0.12	2.38	0.00	0.00	65.10
Control19	3	4001	0.08	1.38	0.00	0.00	47.03
Control20	3	4173	0.06	1.20	0.00	0.00	57.10
Control21	3	5235	0.11	2.25	0.00	0.00	89.00
Control22	3	4091	0.11	2.52	0.00	0.00	94.82
Control23	3	5337	0.11	2.14	0.00	0.00	86.82
RA01	1	5368	0.05	0.58	0.00	0.00	27.53
RA03	1	3498	0.04	0.57	0.00	0.00	16.34
RA04	1	3584	0.05	0.50	0.00	0.00	14.23
RA05	1	4937	0.07	0.85	0.00	0.00	44.25
RA06	1	3983	0.05	0.60	0.00	0.00	27.52
RA07	1	3131	0.05	0.34	0.00	0.00	10.71
RA08	1	6878	0.04	0.41	0.00	0.00	13.57
RA09	2	3520	0.05	0.62	0.00	0.00	30.38
RA10	2	2999	0.05	0.41	0.00	0.00	9.18

RA15	2	4783	0.05	0.81	0.00	0.00	46.52
RA16	3	3135	0.12	1.88	0.00	0.00	63.92
RA17	3	3531	0.05	1.04	0.00	0.00	41.09
RA18	3	2912	0.03	0.53	0.00	0.00	24.09
RA19	3	3699	0.05	0.81	0.00	0.00	29.65
RA20	3	3427	0.10	1.89	0.00	0.00	82.81
RA21	3	3213	0.11	2.43	0.00	0.00	91.97
RA22	3	4448	0.11	2.16	0.00	0.00	66.05
RA23	3	5051	0.09	1.61	0.00	0.00	64.84

Table S8 | Cell counts per sample, before and after preprocessing and filtering.

Sample	Batch	Raw data	RA Samples Only	After removing doublets	After Filtering
Control01	1	3613	3613	2690	2057
Control02	1	4154	Removed matching sample	Removed matching sample	Removed matching sample
Control03	1	3578	3578	2735	2334
Control04	1	6465	6465	5049	4084
Control05	1	7564	7564	6021	4646
Control06	1	3134	3134	2641	1937
Control07	1	6185	6185	4280	3333
Control07	2	4701	4701	3052	2568
Control07	3	10677	10677	7856	4893
Control08	1	6282	6282	5365	3818
Control09	2	8003	8003	6509	5035
Control10	2	8952	8952	6428	5018
Control11	2	6381	Removed matching sample	Removed matching sample	Removed matching sample
Control12	2	1997	Removed matching sample	Removed matching sample	Removed matching sample
Control13	2	4377	Removed matching sample	Removed matching sample	Removed matching sample
Control14	2	5532	Removed matching sample	Removed matching sample	Removed matching sample
Control15	2	6175	6175	5068	4138
Control16	2	7112	7112	5503	4871
Control17	3	3938	3938	3154	1758
Control18	3	4574	4574	3283	1518
Control19	3	6171	6171	4988	2816
Control20	3	6143	6143	5354	3048
Control21	3	9061	9061	6936	4112
Control22	3	5921	5921	5082	3152
Control23	3	8665	8665	6685	4348

RA01	1	89	NA	NA	NA
RA02	1	6091	6091	5562	4250
RA03	1	163	Removed due to low count	Removed due to low count	Removed due to low count
RA04	1	5138	5138	3596	2999
RA05	1	5171	5171	3672	3111
RA06	1	7489	7489	5158	4034
RA07	1	4700	4700	4131	3097
RA08	1	3882	3882	3256	2487
RA09	2	9015	9015	7092	5343
RA10	2	4256	4256	3596	2904
“RA11”	2	3443	3443	3072	2720
“RA12”	2	5170	Not relevant sample	Not relevant sample	Not relevant sample
“RA13”	2	7668	Not relevant sample	Not relevant sample	Not relevant sample
“RA14”	2	7000	Not relevant sample	Not relevant sample	Not relevant sample
RA15	2	7044	Not relevant sample	Not relevant sample	Not relevant sample
RA16	3	7044	7044	4878	4003
RA17	3	4857	4857	3819	2066
RA18	3	5257	5257	4379	2250
RA19	3	4396	4396	3696	2354
RA20	3	5595	5595	4888	3019
RA21	3	5045	5045	4480	2781
RA22	3	4388	4388	3878	2225
RA23	3	6922	6922	5451	3110
nan	1	53	NA	NA	NA
nan	2	26	NA	NA	NA
nan	3	10	NA	NA	NA
**Mean (SD)**	**NA**	**5400.8 (2348.6)**	**5990.7 (1842.8)**	**4731.5 (1376.0)**	**3307.8 (1031.7)**
**Total**	**NA**	**275442**	**227647**	**179797**	**125698**

Table S9 | Cell counts per lane, before and after preprocessing and filtering. SD: Standard deviation.

Lane	Original data	RA Samples Only	After removing doublets	After filtering
run1-1	19865	18853	15052	11753
run1-2	20233	19078	15228	11973
run1-3	22690	21587	16078	12166
run1-4	19889	18789	14890	11638
run2-1	23474	12509	9824	7853
run2-2	22481	12079	9352	7720
run2-3	24583	13170	10210	8144
run2-4	22563	11928	8720	7540
run3-1	23363	23363	18907	11328
run3-2	25618	25617	20631	12227
run3-3	25393	25391	20364	11620
run3-4	25290	25283	20541	11736
**Mean (SD)**	**22953.5 (2097.2)**	**18970.6 (5426.6)**	**14983.1 (4560.2)**	**10474.8 (1984.0)**
**Total**	**275442**	**227647**	**179797**	**125698**

Table S10 | Number of cells within each cell subset. CD: Cluster Differentiation; DCs: Dendritic Cells; IFIT: Interferon Induced proteins with Tetratricopeptide repeats; IFITM: Interferon-induced Transmembrane proteins; IL: Interleukin; IFN: Interferon; NK: Natural Killer; Tem: T Effector Memory; TEMRA: Terminally Differentiated Effector Memory.

**Subset**	**Count**
CD4 T central memory	21531
CD4 T effector memory	12531
CD4 T IFIT	595
CD4 T Naive	17043
Gamm-delta T cells	1424
CD8 T early Tem	2292
CD8 T Naive	2882
CD8 TEMRA	2702
Classical Monocytes	9110
IFITM3+ IFN-Activated Monocytes	581
IL1b-Monocytes	5700
Myeloid DCs	6047
Non-classical Monocytes	2171
NK CD56bright	7676
NK CD56low	2022
Memory Bcells	6177
Naive Bcells	6760
Plasmablasts	1473
